# PfHDAC1 is an essential regulator of *P. falciparum* asexual proliferation and host cell invasion genes with a dynamic genomic occupancy responsive to artemisinin stress

**DOI:** 10.1128/mbio.02377-23

**Published:** 2024-05-06

**Authors:** Abhishek Kanyal, Bhagyashree Deshmukh, Heledd Davies, D. V. Mamatharani, Dilsha Farheen, Moritz Treeck, Krishanpal Karmodiya

**Affiliations:** 1 Department of Biology, Indian Institute of Science Education and Research, Dr. Homi Bhabha Road, Pashan, Pune, Maharashtra, India; 2 Signalling in Apicomplexan Parasites Laboratory, The Francis Crick Institute, London, United Kingdom; Duke University School of Medicine, Durham, North Carolina, USA

**Keywords:** malaria, *Plasmodium falciparum*, histone deacetylase, PfHDAC1, transcriptomics, artemisinin resistance

## Abstract

*Plasmodium falciparum,* the deadly protozoan parasite responsible for malaria, has a tightly regulated gene expression profile closely linked to its intraerythrocytic development cycle. Epigenetic modifiers of the histone acetylation code have been identified as key regulators of the parasite’s transcriptome but require further investigation. In this study, we map the genomic distribution of *Plasmodium falciparum* histone deacetylase 1 (PfHDAC1) across the erythrocytic asexual development cycle and find it has a dynamic occupancy over a wide array of developmentally relevant genes. Overexpression of PfHDAC1 results in a progressive increment in parasite load over consecutive rounds of the asexual infection cycle and is associated with enhanced gene expression of multiple families of host cell invasion factors (merozoite surface proteins, rhoptry proteins, etc.) and with increased merozoite invasion efficiency. With the use of class-specific inhibitors, we demonstrate that PfHDAC1 activity in parasites is crucial for timely intraerythrocytic development. Interestingly, overexpression of PfHDAC1 results in decreased sensitivity to frontline-drug dihydroartemisinin in parasites. Furthermore, we identify that artemisinin exposure can interfere with PfHDAC1 abundance and chromatin occupancy, resulting in enrichment over genes implicated in response/resistance to artemisinin. Finally, we identify that dihydroartemisinin exposure can interrupt the *in vitro* catalytic deacetylase activity and post-translational phosphorylation of PfHDAC1, aspects that are crucial for its genomic function. Collectively, our results demonstrate PfHDAC1 to be a regulator of critical functions in asexual parasite development and host invasion, which is responsive to artemisinin exposure stress and deterministic of resistance to it.

**IMPORTANCE:**

Malaria is a major public health problem, with the parasite *Plasmodium falciparum* causing most of the malaria-associated mortality. It is spread by the bite of infected mosquitoes and results in symptoms such as cyclic fever, chills, and headache. However, if left untreated, it can quickly progress to a more severe and life-threatening form. The World Health Organization currently recommends the use of artemisinin combination therapy, and it has worked as a gold standard for many years. Unfortunately, certain countries in southeast Asia and Africa, burdened with a high prevalence of malaria, have reported cases of drug-resistant infections. One of the major problems in controlling malaria is the emergence of artemisinin resistance. Population genomic studies have identified mutations in the Kelch13 gene as a molecular marker for artemisinin resistance. However, several reports thereafter indicated that Kelch13 is not the main mediator but rather hinted at transcriptional deregulation as a major determinant of drug resistance. Earlier, we identified PfGCN5 as a global regulator of stress-responsive genes, which are known to play a central role in artemisinin resistance generation. In this study, we have identified PfHDAC1, a histone deacetylase as a cell cycle regulator, playing an important role in artemisinin resistance generation. Taken together, our study identified key transcriptional regulators that play an important role in artemisinin resistance generation.

## INTRODUCTION

Malaria is a deadly disease that affects roughly 247 million people around the world and is responsible for close to 619,000 deaths annually, mostly in the under-privileged developing countries (1). Among the five species that infect humans, *Plasmodium falciparum* is associated with maximum morbid manifestations and takes the lead in death tolls by the parasite (2). Over the years, a plethora of therapeutic agents have been utilized with initial success to curb this disease, but the parasite has shown resilience and emerged resistant, to varying degrees, to most of these pharmaceutical drugs (3). Artemisinin-based combination therapy despite being tremendously effective in clearing parasite load is threatened in the face of emerging artemisinin resistance across the world (4). However, *Plasmodium falciparum*, bolstered by mutations in key genes [including PfKelch13 (PF3D7_1343700) and PfCoronin (PF3D7_1251200)], and changes to lipid and heme metabolism have emerged resistant to artemisinin gradually, especially in the Greater Mekong Subregion of southeast Asia (5–8). Comparative multi omics profiling of artemisinin-resistant parasites has revealed substantial reconfiguration of gene expression networks in response to the drug to reduce its activation and enhance the mitigatory homeostatic functions in parasites (9, 10). This highlights the need to uncover these regulatory and adaptive mechanisms in the parasite.

*Plasmodium falciparum* displays a strictly coordinated expression of key genes over the 48-hour intraerythrocytic development cycle (IDC) where it transitions through phases of increasing metabolic activity, replication, culminating in optimal utilization of host nutrients, and then egressing to infect a fresh set of cells (11, 12). Characterized by relatively AT-rich genomes, multiple *Plasmodium* species have come to utilize a host of epigenetic mechanisms (especially histone variants and modifications) to orchestrate gene expression regulation (13, 14). Modifiers (and their complexes) of the histone acetylation code in *P. falciparum* are correlated with parasite transcription, respond to physiological stresses (fever cycles), and regulate antigenic variation and virulence (15–17). Due to their sequence proximity to counterpart molecules in earlier eukaryotes, the histone acetyltransferase and deacetylase have been heavily investigated as potential therapeutic targets for epigenetic modulators and inhibitors. Notably, histone deacetylase inhibitors adapted from anti-cancer drug panels using piggy-back approach have shown great promise with their anti-*Plasmodium* activity (18). Despite their targetability, relatively little is known about the histone deacetylases themselves.

PfHDAC1 is the “lone-wolf” of the *Plasmodium falciparum* class I HDACs and is still very poorly studied. Genome-wide mutagenesis screens have suggested the gene to be essential in both *P. falciparum* and *P. berghei* (19, 20). This underlines the potentially critical role HDAC1 plays in *Plasmodium* biology. Numerous drugs [including entinostat (MS-275), romidepsin, valproic acid, LMK-35, and NSAID] with potent anti-parasite activity are believed to operate via targeting the PfHDAC1 (18, 21–24). HDAC1 in higher order eukaryotes has been associated with regulating key biological functions in tandem with chromatin remodeling associated with regulation of cell cycle, developmental fates, response to therapeutic pressure, DNA replication/damage repair, autophagy, and protein quality control (25–30). It is quite likely that PfHDAC1 occupies a similar important place in gene regulatory functions in *P. falciparum*. Interestingly, a population transcriptomics study conducted on artemisinin-resistant and -sensitive parasites from southeast Asia by Mok et al. has identified PfHDAC1 as one of the most significantly deregulated genes (31). Given the evidential role of HDAC deregulation in emergence of chemotherapeutic resistance in mammalian systems, it becomes highly interesting to cross-examine the role of PfHDAC1 in governing resistance to antimalarial compounds (chiefly artemisinin) against *P. falciparum* (32, 33).

In this study, we characterize the genomic occupancy and gene regulatory role of PfHDAC1. Using ChIP-sequencing, we identify the dynamic set of PfHDAC1 gene targets across the parasite asexual cycle and regulation of the biological functions they are linked to. We further investigate the effect of alteration of PfHDAC1 abundance and activity on the asexual proliferation and infection progression in parasites. We demonstrate that PfHDAC1 overexpression is associated with enhanced resistance to dihydroartemisinin (DHA) drug and examine the effect of the drug in modulating the genomic occupancy of PfHDAC1 in wild-type parasites. Additionally, we discover that dihydroartemisinin treatment can interfere with the catalytic deacetylase activity and phosphorylation post-translational modification of PfHDAC1.

## RESULTS

### PfHDAC1 shows a dynamic genomic occupancy and targets a wide range of gene/functions with stage-specific expression in parasites

**Fig 1:**
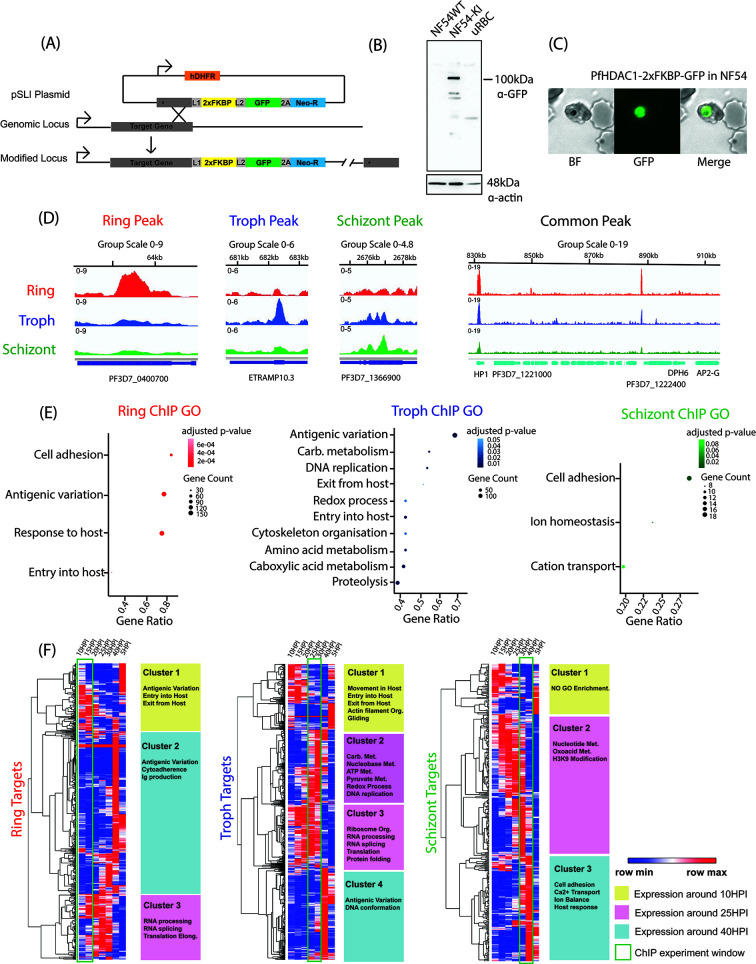
PfHDAC1 dynamically binds to stage-specific *Plasmodium* genes associated with house-keeping as well as stress-responsive functions. (**A**) Schematic of the PfHDAC1-2×FKBP-GFP knockin construct and recombination/integration into the genomic locus. (**B**) Western blotting (α-GFP) on NF54 wild-type control (NF54WT), PfHDAC1-2×FKBP-GFP knockin (NF54-KI), and uninfected RBC control (uRBC) lines and (**C**) confocal microscopy (α-GFP) in NF54-KI line to validate the knockin (representative of two biological replicates). (**D**) IGV browser sample shots of tracks displaying stage-specific PfHDAC1 enrichment peaks in ring [10 hours post invasion (HPI)], trophozoite (24 HPI), and schizont (40 HPI) stages and two sample peaks shared across IDC. (**E**) Dotplots representing GO enrichment of biological pathways underlying PfHDAC1 ChIP target genes in ring, trophozoite, and schizont stages (left to right). All figures for ChIP-seq are representative of two biological replicates. (**F**) Gene expression matrices of PfHDAC1 ChIP targets identified in ring (left), trophozoite (middle), and schizont (right). Expression of target genes is plotted for seven-time intervals covering a full IDC (10 to 5 HPI/cycle 2). Clusters 1–4 with heightened PfHDAC1 target gene expression window around ring (yellow), trophozoite (pink), and schizont (teal) stages are highlighted with associated biological pathways mentioned. The time-window of ChIP experiment is demarcated in green box.

Although PfHDAC1 has been described as a target of multiple highly potent inhibitors, a thorough investigation into its genomic occupancy is lacking in *P. falciparum* (18, 34–36). We first sought to identify the genome-wide targets of PfHDAC1 across the ring, trophozoite, and schizont stage of the intraerythrocytic development cycle. We generated a transgenic NF54 strain parasite line in which the endogenous PfHDAC1 was tagged with 2×FKBP-GFP at the C-terminus (Fig. 1A) (adapted from reference 37). We validated the GFP-tagged PfHDAC1 using α-GFP antibody by western blotting (Fig. 1B) and using confocal microscopy (Fig. 1C). Additional validation of the GFP knockin line was performed by integration-specific PCR amplification and inspection of next-generation sequencing data from the edited locus (Fig. S1A and B). Immunofluorescence and western blotting revealed very weak expression of PfHDAC1 in ring-stage parasites which later became robust in trophozoite and schizont stages (Fig. S1C and D). Moreover, the PfHDAC1 was predominantly localized to the parasite nuclei.

We performed ChIP-sequencing in ring [10 hours post invasion (HPI)], trophozoite (24 HPI), and schizont (40 HPI) stage parasites using α-GFP antibody (Tables S1 and S2). DNA sequenced from pre-IP-sonicated chromatin was taken as input for normalization and peak calling. We identified 1,003 peaks (over 567 genes) for PfHDAC1 chromatin occupancy in rings, 2,108 peaks (over 1,393 genes) in 24 HPI trophozoites, and 450 peaks (over 366 genes) for schizonts (all peaks ≥twofold enriched in ChIP over input) (listed in Table S2; sampled in Fig. 1D), indicating at a variable genomic occupancy of PfHDAC1 throughout the IDC. The overall chromatin occupancy profile of PfHDAC1 was distinct across the ring, trophozoite (also sampled at 21 HPI for a later comparison), and schizont stages with a few target genes still shared across stages (Fig. S2A and B). Gene ontology analysis on the target genes revealed enrichment of a wide variety of biological processes. Ring-stage PfHDAC1 targets enriched for antigenic variation, cytoadherence, and entry into host cells primarily (Fig. 1E, left). This stage reports modest transcription overall and reflects a persistence of invasion- and virulence-associated genes (38). The trophozoite-stage targets were enriched for a wider range of biological functions, including antigenic variation, carboxylic acid metabolism, DNA replication, carbohydrate metabolism, proteolysis, entry and exit into/from host cell, pre-replicative complex assembly, ATP metabolism, redox process, and hemoglobin metabolism (Fig. 1E, middle). These are all well-reported functions associated with trophozoites stage development of the parasite (11, 39). Finally, schizont-stage parasites had a reduced repertoire of gene targets, and associated biological processes mainly impinged on cellular adhesion and ion homeostasis (Fig. 1E, right). Schizont-stage parasites, in general, are characterized by preparation to egress and initiate fresh round of infection (regulated by ion signaling) (40).

Furthermore, we plotted the expression profile for the PfHDAC1 gene targets of each IDC stage (R/T/S), using published RNA seq data set across the IDC (41). We did not identify any global suppression of target genes associated with PfHDAC1 occupancy in a particular stage. In fact, target gene sets in each stage could be classified into three to four clusters of highly or lowly expressed genes relative to other stages of IDC. In ring stage, we identified three clusters of target gene expression status: cluster 1, gene with high gene expression in the 10–15 HPI window but repressed later on (enriched for entry and exit into/from host cell, antigenic variation); cluster 2, genes with low expression in 10–15 HPI window and expressed only later (antigenic variation, immunoglobin production); cluster 3, genes with modest expression in 10–15 HPI increasing later in trophozoite stage (RNA splicing and translation) (Fig. 1F, left). In trophozoite-stage PfHDAC1, targets could be segregated as: cluster 1, expressed early but shut down around 25–30 HPI (entry and exit from host cell, actin organization, gliding); clusters 2 and 3, expressed highly in 25–30 HPI window (carbohydrate metabolism, redox, DNA replication, RNA processing); cluster 4, suppressed in 25–30 HPI but evoked later (antigenic variation, DNA conformation) (Fig. 1F, middle). Finally, in the schizont stage, we could trace target gene expression classifiable into clusters 1 and 2, expressed earlier but shut down at 40 HPI (nucleotide/oxo-acid metabolism); cluster 3, expressed sharply in 40 HPI window (Ca^2+^ ion transport, ion balance, cell adhesion) and repressed otherwise (Fig. 1F, right).

Thus, we found that PfHDAC1 genomic occupancy closely follows the major biological activity demonstrated by the parasites across the IDC. Our data suggest the enrichment of PfHDAC1 over a wide variety of crucial stage-specific biological processes across the IDC in the parasites. Throughout the IDC, the association of PfHDAC1 with its target genes seemingly allows for their timely expression and shut down. We observed an overall positive association of PfHDAC1 occupancy with expression of host cell entry/egress genes, RNA splicing, energy metabolism, DNA replication genes, and a suppressive influence over antigenic variation-associated genes. The enrichment of PfHDAC1 targets peaked and diversified, especially during trophozoite stage of IDC (a stage which is characterized by the transcriptional burst of genes from a wide array of biological functions). Further work would be required to dissect the control of PfHDAC1 over stage-specific gene expression in the parasite.

### PfHDAC1 overexpression is associated with a proliferation advantage in parasites

**Fig 2:**
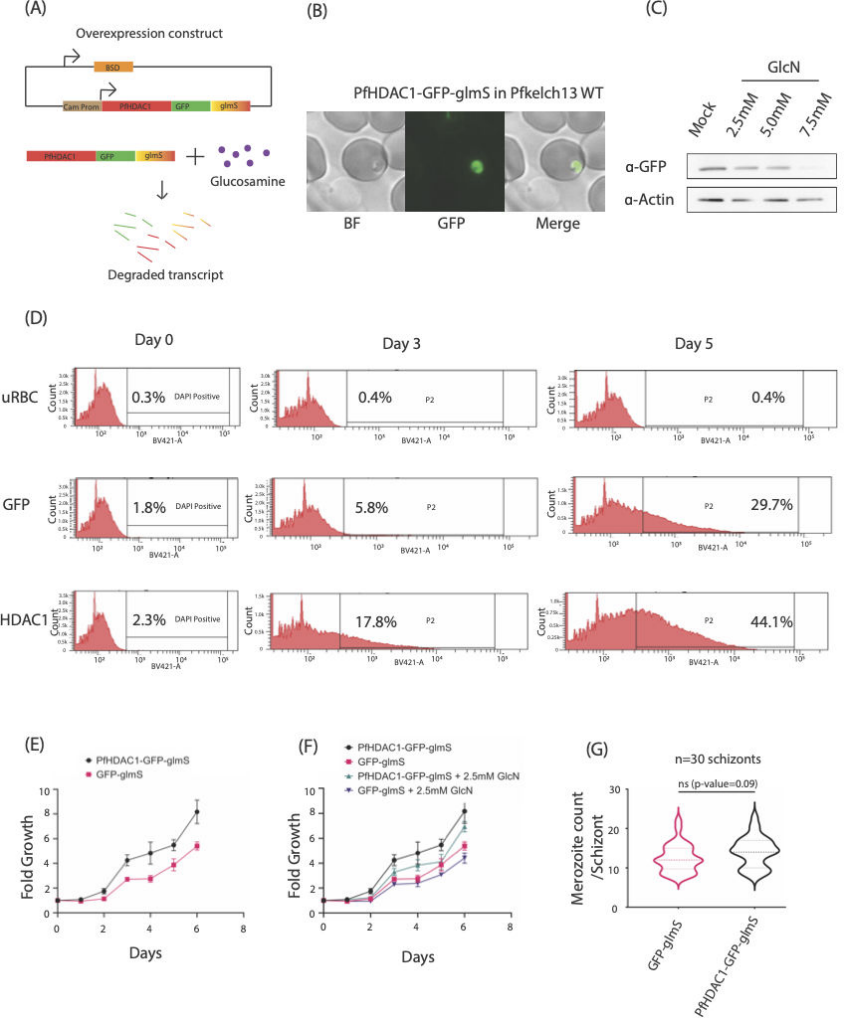
PfHDAC1 overexpression is associated with a proliferative advantage in parasites. (**A**) Schematic of the pDC2 plasmid used for calmodulin promoter-driven episomal overexpression of PfHDAC1-GFP-glmS. The ribozyme-tagged transcript can be degraded upon addition of glucosamine ligand. (**B**) Confocal microscopy-based validation of PfHDAC1-GFP-glmS expression. (**C**) Western blotting-based validation of PfHDAC1-GFP-glmS overexpression in parasite lysate and its progressive depletion with dosed glucosamine treatment (0, 2.5, 5, 7.5 mM GlcN-HCl). Actin probing is used as loading control. Blot is representative of three biological replicates. (**D**) Flow cytometry histogram curves for DAPI^+^ (infected) gated population across 5-day parasite growth assay. Top panel is for uninfected RBCs, middle for GFP-glmS overexpression parasite-infected RBCs, and bottom for PfHDAC1-GFP-glmS overexpression parasites. (**E**) Line plot to demonstrate the growth advantage observed in PfHDAC1-GFP-glmS overexpression parasites compared to control GFP-glmS overexpression line. The data are representative of PfKelch13WT genotype parasites. (**F**) Line plot representing the growth of PfHDAC1-GFP-glmS and GFP-glmS parasite lines under mock vs 2.5 mM glucosamine treatment. Fold growth is quantified as fold increase in parasitemia over the starting (day 0) parasitemia. Parts E–F are representative of one biological replicate and three technical replicate readouts at each timepoint. (**G**) Violin plot representing the merozoite counts from GFP-glmS and PfHDAC1-GFP-glmS overexpression parasites recorded from ripe-segmented 44 HPI schizonts (*n* = 30 schizonts). Plot is representative of two biological replicates.

Our assessment of PfHDAC1 gene targets revealed enrichment over processes linked to parasite development and progression of infection. We decided to investigate the effect of PfHDAC1 overexpression on intra-erythrocytic development and proliferation in RBCs. We episomally overexpressed a GFP-tagged PfHDAC1 (driven by the calmodulin promoter) in the MRA-1252 parasite strain. Additionally, the glmS ribozyme sequence was introduced downstream of the PfHDAC1-GFP sequence to allow for tunable overexpression using glucosamine ligand (Fig. 2A). The overexpression lines were confirmed with α-GFP confocal microscopy (Fig. 2B). Also, with increasing glucosamine treatment (0–7.5 mM), we observed a progressive depletion of the protein using western blotting (Fig. 2C). A GFP-glmS overexpression line was used as a control.

We first investigated the effect of PfHDAC1 overexpression on parasite proliferation using flow cytometry. Uninfected RBC control, GFP-glmS control, and PfHDAC1-GFP-glmS overexpression parasite lines were followed over a course of 5 days to assess their proliferation dynamics (Fig. 2D). The experiment commenced with uRBC, GFP-glmS, and PfHDAC1-GFP-glmS overexpression cultures reporting 0.3%, 1.8%, and 2.3% DAPI-positive population, respectively (day 0 timepoint) (Fig. 2D, top). The DAPI-positive population in uRBC control culture stayed steadily at 0.4% on day 3 and day 5 post invasion. while in GFP-glmS, culture rose to 5.8% on day 3 post invasion and 29.7% on day 5 post invasion, establishing a baseline proliferation rate (Fig. 2D, middle). The DAPI-positive population in PfHDAC1-GFP-glmS overexpression culture rose to 17.8% on day 3 post invasion and 44.1% on day 5 post invasion (Fig. 2D, bottom). Thus, we observed an increase in parasite proliferation rate for PfHDAC1-GFP-glmS overexpression line as compared to the GFP-glmS overexpression control. We next performed a 6-day parasite growth assay testing for effect of tunable PfHDAC1-GFP-glmS overexpression using mock and 2.5 mM glucosamine. Parasite growth under mock or 2.5 mM glucosamine treatment in control (GFP-glmS overexpression) and test (PfHDAC1-GFP-glmS overexpression) lines was recorded every 24 hours. This was plotted as a fold change relative to their starting day 0 parasitemia. The parasitemia in the PfHDAC1 overexpression line was observed to have higher fold growth than the GFP control line with every cycle under mock treatment (Fig. 2E). This growth advantage was, however, observed to be lesser in glucosamine treatment setup (Fig. 2F). Thus, even minor depletion of the overexpressed PfHDAC1 by glucosamine was found to be associated with loss of growth benefits. Furthermore, growth assays carried out on the PfHDAC1-GFP-glmS overexpression parasites with a graded treatment of glucosamine (mock, 2.5, 5, and 7.5 mM) revealed a sharp drop in parasite growth under treatment by two subsequent reinvasion cycles (Fig. S2C).

We then tested if the overexpression of PfHDAC1 was associated with changes in progression of the cell cycle across 48 hours. IDC by sampling parasites from tightly synchronized PfHDAC1-GFP-glmS overexpression line and GFP-glmS overexpression control line at 8-hour intervals starting 10 hours post invasion. No significant changes in morphology or shift in phases of development were observed in PfHDAC1 overexpression vs control line (Fig. S2D). We subsequently tested if an increased proliferation could be a consequence of increased merozoite counts. We counted the number of merozoites in mature segmented schizonts at 46 HPI in tightly synchronized GFP-glmS overexpression lines and PfHDAC1-GFP-glmS overexpression lines. We found the merozoite counts to be slightly elevated in PfHDAC1-GFP-glmS overexpression line, but the difference did not reach statistical significance (*P*-value = 0.09) (Fig. 2G; Fig. S2E). Additionally, we detected no change to the merozoite count per schizont using the PfHDAC1-GFP-glmS overexpression line cultured under increasing dosage of glucosamine (0, 2.5, 5.0, and 7.5 mM concentration) (Fig. S2F). Thus, PfHDAC1 overexpression was found to be associated with enhanced proliferation of parasites over subsequent IDCs, albeit without any shift in stage progression or significant change to merozoite count.

### PfHDAC1 overexpression is associated with enhanced expression of multiple families of host cell invasion-associated genes and increased merozoite invasion phenotype

**Fig 3:**
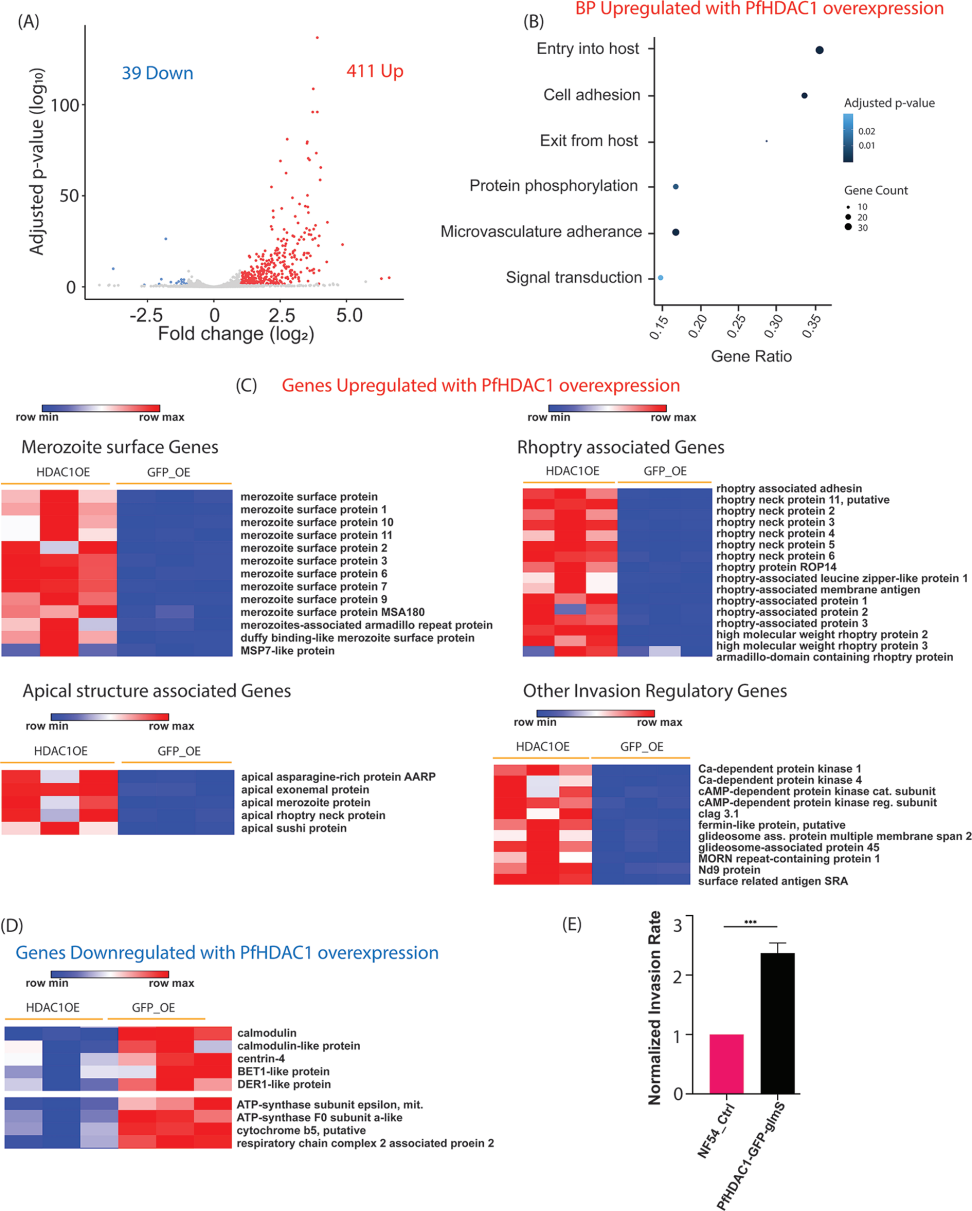
PfHDAC1 overexpression is associated with enhanced expression of multiple families of host cell invasion-associated genes in parasites. (**A**) Volcano plot representing the differentially expressed genes in PfHDAC1-GFP-glmS overexpression parasites compared to GFP-glmS overexpression control parasites at ~24 HPI. (**B**) Dotplot representing the GO-enriched biological processes upregulated with PfHDAC1 overexpression. (**C**) Heatmaps of genes from various host cell invasion-associated parasite gene families which are upregulated in PfHDAC1-GFP-glmS overexpression parasite line. (**D**) Heatmap of selected Ca^2+^ signaling and nuclear-encoded mitochondrial ETC-associated genes downregulated with PfHDAC1-GFP-glmS overexpression. All data are representative of three biological replicate RNA sequencing. (**E**) Histograms representing the NF54 control normalized merozoite invasion rate noted for NF54 and PfHDAC1-GFP-glmS overexpression line. Unpaired *t*-test used for statistics. The data are representative of three biological replicates.

To gain insight into the transcriptional consequences of PfHDAC1 abundance in cells (and if these could explain the proliferation benefits), we compared PfHDAC1-GFP-glmS overexpression vs GFP-glmS control overexpression parasites at 24 HPI (early trophozoite) using strand-specific RNA sequencing (performed in biological triplicate) (Tables S1 and S3). We identified PfHDAC1 among one of the top genes upregulated (log2 fold change of 2.17; *P*-adjusted: 1.49e−55) in the episomal overexpression parasite lines. Interestingly, PfHDAC1 overexpression caused more genes to be upregulated (411 genes) than downregulated (39 genes) (with log2 fold change ≥−1/1; *P*-value ≤0.05), indicating a possible indirect effect of PfHDAC1 overexpression on transcription (Fig. 3A). Gene ontology analysis of upregulated genes upon PfHDAC1 episomal overexpression revealed enrichment of genes for entry into host cell (rhoptry proteins, 6-cysteine proteins, merozoite surface protein), cytoadherence to microvasculature (EMP, rifin, and stevor), immunomodulators [SERA 1, 3, 4, and 5 (PF3D7_0208000, PF3D7_0207900, PF3D7_0207800, and PF3D7_0207700, respectively)], protein phosphorylation/signaling cascade (especially FIKK proteins), and exit from host cell (Fig. 3B). Genes associated with host cell invasion/egress included merozoite surface protein [msp 1, 3, and 7 (PF3D7_0930300, PF3D7_1035400, and PF3D7_1335100, respectively)], rhoptry neck protein [ron 3, 4, and 6 (PF3D7_1252100, PF3D7_1116000, and PF3D7_0214900, respectively)], and high molecular weight rhoptry protein [rhoph2 and rhoph3 (PF3D7_0929400 and PF3D7_0905400)] whose products help the egressed parasites to bind onto fresh host RBCs during re-invasion (Fig. 3C) (42, 43). A Ca^2+^-dependent protein kinase 1 (PF3D7_0217500), cyclic-AMP-dependent protein kinase catalytic and regulatory subunit (PF3D7_0934800, PF3D7_1223100), surface-related antigen SRA (PF3D7_1431400) were among the genes strongly upregulated with PfHDAC1 overexpression (Fig. 3C). These genes have been characterized for their role in parasite invasion of RBCs (44–46).

Among the 39 downregulated genes, we did not find a strong enrichment for any biological process. We did note downregulation of three Ca^2+^-mediated signaling genes centrin 4 (PF3D7_1105500), calmodulin (PF3D7_1434200), and a calmodulin-like gene (PF3D7_0414200) (Fig. 3D). Additionally, four nuclear-encoded genes associated with mitochondrial function were found to be downregulated with PfHDAC1 overexpression. These included the ATP-synthase F_0_ subunit a-like protein (PF3D7_0719100), ATP-synthase epsilon subunit (PF3D7_0715500), respiratory chain complex 2-associated protein 2 (PF3D7_1322800), aminomethyltransferase (PF3D7_1452200), and a putative cytochrome b5 (PF3D7_1232300) (Fig. 3D). To confirm whether PfHDAC1-GFP-glmS overexpression could drive enhanced merozoite invasion, we performed an invasion assay which revealed increased merozoite invasion rates in PfHDAC1-GFP-glmS overexpression line compared to NF54 control line (2.37-fold increase; standard deviation: 0.17) (Fig. 3E). Thus, episomal overexpression of PfHDAC1 was found to be associated with an upregulation of a wide variety of RBC invasion-associated genes and an enhanced invasion phenotype in merozoites. This could explain the phenotypic observation of increased parasitemia over consecutive IDC despite no shift in the IDC stage and merozoite count in schizonts.

**Fig 4:**
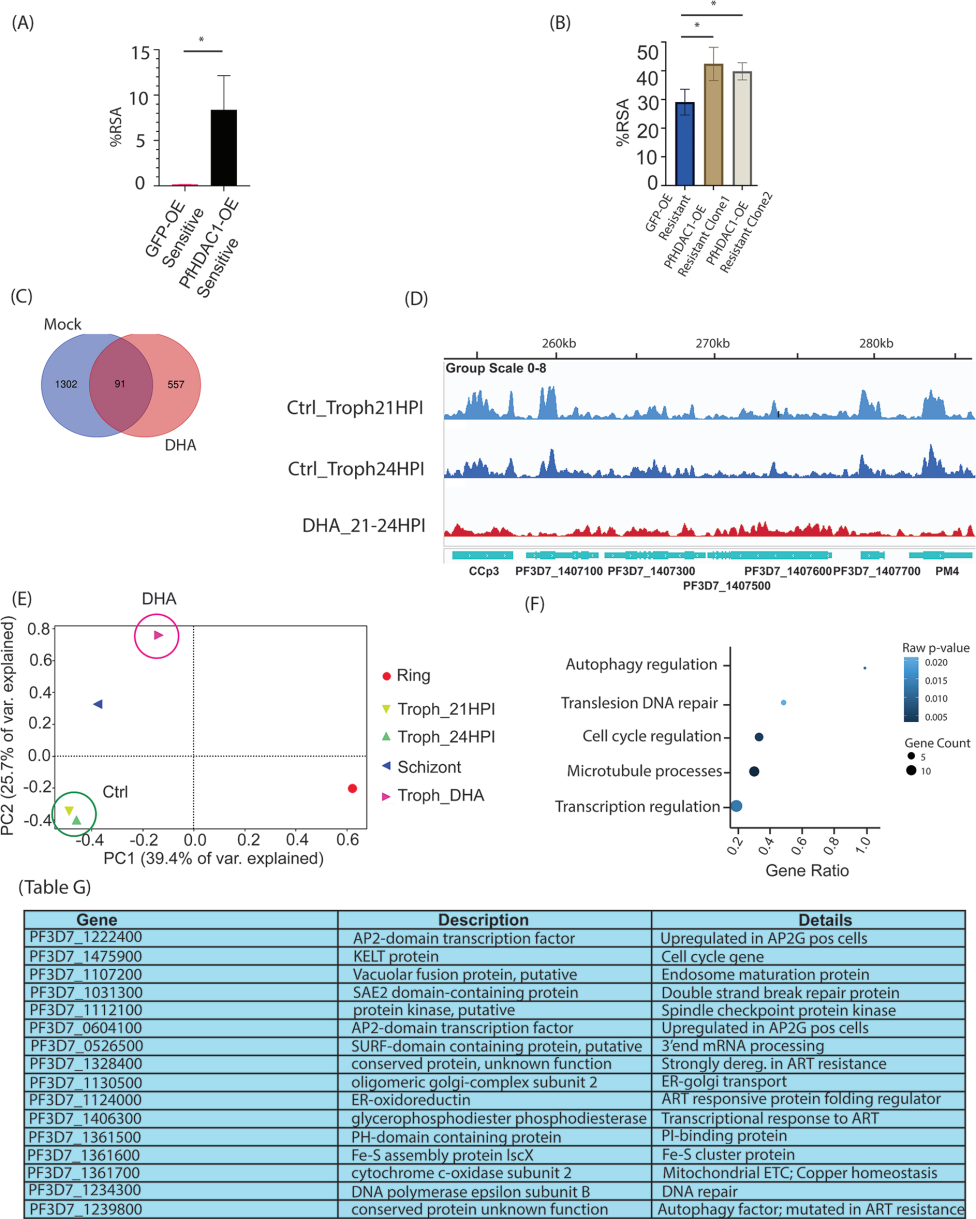
PfHDAC1 overexpression enhances dihydroartemisinin resistance in parasites, while the drug exposure interferes with and reorganizes the genomic occupancy of PfHDAC1. (**A**) Barplot representing the %RSA of GFP-glmS control overexpression and PfHDAC1-GFP-glmS overexpression lines generated with PfKelch13-WT parasite line (MRA-1252 strain) (statistical analysis used: Student’s unpaired *t*-test; * represents *P*-value 0.019). (**B**) Barplot representing the % RSA of one GFP-glmS control overexpression and two PfHDAC1-GFP-glmS overexpression lines generated with PfKelch13-R539T mutant parasite line (MRA-1240 strain). PfHDAC1-GFP-glmS overexpression lines report elevated %RSA (enhanced artemisinin resistance) when compared with cognate GFP-glmS control lines (statistical analysis used: Student’s unpaired *t*-test; * represents *P*-value 0.034 for clone 1 and *P*-value 0.026 for clone 2). (**C**) Venn diagram displaying PfHDAC1 target gene counts in control vs dihydroartemisinin-treated parasites. (**D**) Sample shots of PfHDAC1 ChIP-seq peaks in 21 and 24 HPI trophozoites vs dihydroartemisinin-treated trophozites at 24 HPI (captured in IGV browser). Parts C and D represent ChIPseq from two biological replicates each of 21 and 24 HPI trophozoites and 24 HPI DHA-treated parasites (100 nM DHA for 3 hours/21–24 HPI). (**E**) PCA plot depicting the correlation of input-normalized ChIP data set from the ring, trophozoite (21 and 24 HPI), and schizont timepoints with the DHA-treated 24 HPI trophozoite. (**F**) Dotplot representing the GO-enriched biological processes for PfHDAC1 target genes in DHA exposure. (Table G) Table representing the top PfHDAC1 target genes under DHA exposure and their attributes. ChIP data set represents merged results from two biological replicates for each datapoint.

As we observed enhanced proliferation and invasion with PfHDAC1 overexpression, we next performed chemical inhibition of it using sublethal doses of romidepsin (Supplementary Material and Fig. S3A through C). Importantly, PfHDAC1 inhibition was shown to delay cell cycle progression compounded with defects in proper morphological development of parasites and reduction in proliferation over to the next cycle of the intraerythrocytic development (Fig. S3A through C). Interestingly, treatment of parasites with 100 nM DHA resulted in depletion of PfHDAC1 protein levels, hinting at a direct effect of the antimalarial on it (Supplementary Methods and Fig. S3D). Additionally, we investigated the transcriptional changes associated with the PfHDAC1 inhibitor treatment using RNA sequencing (Fig. S3E through G and Fig. 4A; Tables S1 and S3). We further corroborated these data with the existing data from PfHDAC1 genetic depletion and RNA sequencing by Huang et al. (34) (Supplementary Material and Fig. S4A through C; Table S4) (34). Taken together, these observations highlight the importance of PfHDAC1 activity in timely progression of the parasite IDC, optimal DNA replication, and passage of infection to the consecutive cycle.

### PfHDAC1 overexpression is associated with decreased sensitivity to dihydroartemisinin

Dihydroartemisinin is a frontline drug effective against multidrug-resistant *P. falciparum*. Previous studies have demonstrated the efficacy of pharmacological inhibition of PfHDAC1 in suppressing survival of dihydroartemisinin-resistant parasites. We sought to investigate the effect of PfHDAC1 overexpression on parasite dihydroartemisinin sensitivity using a standard ring-stage survival assay. PfHDAC1-GFP-glmS and GFP-glmS overexpression were achieved in *Plasmodium falciparum* MRA1240 (PfKelch13R539T) and MRA1252 (PfKelch13R539WT) which are resistant and sensitive to artemisinin (47). GFP-glmS-overexpressing MRA-1252 parasites registered a baseline 0.17% RSA, while PfHDAC1-GFP-glmS-overexpressing MRA-1252 parasites registered an increase to 8.45% RSA, indicating rise in dihydroartemisinin resistance associated with PfHDAC1 overexpression (Student’s unpaired *t*-test; * represents *P*-value 0.019) (Fig. 4A).

Furthermore, the RSA for GFP-glmS overexpression MRA-1240 parasites was recorded at 29.05% RSA. We tested the two individual clones of PfHDAC1-GFP-glmS-overexpressing MRA-1240 parasites (clone 1 and clone 2) for their dihydroartemisinin sensitivity. The first PfHDAC1 overexpression clone reported an RSA% of 42.38%, while the second clone recorded an RSA% of 39% (Student’s unpaired *t*-test; * represents *P*-value 0.034 for clone 1 and *P*-value 0.026 for clone 2) (Fig. 4B). Thus, we observed PfHDAC1 overexpression to be associated with an enhanced resistance to dihydroartemisinin in both PfKelch13 R539WT and PfKelch13R539T mutant backdrop, highlighting that PfHDAC1 overexpression in parasites can establish, as well as enhance, pre-existing resistance to artemisinin. While RSA was performed on PfHDAC1-GFP-glmS or GFP-glms overexpression lines and compared them against the identical PfKelch13 wild-type or mutant parasites, we conducted additional validations. Specifically, we scrutinized the PfKelch13 gene locus of the R539 WT and R539T mutant parasite strains employed in RSA to ensure that no other mutations could inadvertently contribute to the observed decrease in artemisinin sensitivity upon episomal PfHDAC1 overexpression. To confirm the specificity of our findings, we analyzed the whole transcriptome data of the resistant lines, revealing the exclusive presence of the R539T mutation, with a notable absence of C580Y and I543T mutations (Fig. S5A and B). Notably, sensitive parasites showed no known PfKelch13 mutations associated with artemisinin resistance. Importantly, the PfKelch13 locus for both PfHDAC1-GFP-glmS and GFP-glmS overexpression lines was found to be identical (Fig. S5A and B). Although we observed additional single-nucleotide polymorphisms (SNPs) in the PfKelch13 loci, none of these were linked to artemisinin resistance and were consistently present across all lines, irrespective of sensitivity or resistance status (Fig. S5C and D). It is possible that overexpression of PfHDAC1 counteracts the effects of the drug in RSA to grant resistance to artemisinin.

### Dihydroartemisinin treatment alters the genomic occupancy of PfHDAC1

Having noted the dihydroartemisinin resistance promoting the effect of PfHDAC1 overexpression, we sought to investigate how the drug influences PfHDAC1 genomic occupancy in wild-type parasites. We performed ChIP-sequencing for PfHDAC1 (using PfHDAC1-2×FKBP-GFP knock-in line) after 3 hours of 100 nM dihydroartemisinin treatment at around 21–24 HPI. DNA sequenced from pre-IP, sonicated chromatin was taken as input for normalization and peak calling. The milder DHA dosage and relatively shorter exposure time were chosen to avoid the consequences of rapid irrecoverable cell death. We found a significant reduction in genomic occupancy of PfHDAC1 upon dihydroartemisinin exposure (648 genes from 676 peaks) as compared to the control condition (1,393 genes from 2,108 peaks) with only a partial overlap between the genomic targets (Fig. 4C and D). Since artemisinin treatment is reported to skew (slow down) the parasite asexual life cycle progression, we investigated if the PfHDAC1 chromatin occupancy captured after 3 hours of DHA treatment (21–24 HPI) was merely occupancy at 21 HPI (due to possible slowing down of the cell cycle); we investigated the chromatin occupancy profile of PfHDAC1 at 21 HPI via ChIP (under no treatment). We identified that the chromatin occupancy profiles of PfHDAC1 at 21 and 24 HPI were highly similar and very distinct from that in DHA-treated parasites (Fig. 4D and E). PfHDAC1 targets under DHA exposure were associated with microtubule-based movement, regulation of cell cycle, regulation of autophagy, and DNA damage repair, albeit the enrichment was not strong (Fig. 4F). A wide majority of pathways under PfHDAC1 were lost or depleted under dihydroartemisinin exposure, including antigenic variation, carboxylic acid metabolism, DNA replication, phosphorylation, and redox homeostasis.

A closer inspection of the top 20 PfHDAC1 targets (by fold-enrichment) revealed hits relevant to parasite response/resistance to artemisinin (Table G in Fig. 4). Of note were two AP2 domain containing transcription factors: PF3D7_1222400, which is reported to be upregulated in AP2G+ cells upon LysoPC depletion; and PF3D7_0604100, which is also upregulated in AP2G+ cells and is listed as a possible regulator of gametocytogenesis (48). There were two DNA damage repair genes among the hits as well, an SAE2 domain containing double-strand break repair protein (PF3D7_1031300) and DNA polymerase epsilon subunit B (PF3D7_1234300). We further identified two genes that have been reported to be perturbed upon artemisinin treatment as targets of PfHDAC1 under DHA stress. These included an Fe-S assembly protein (PF3D7_1361600) (protein slightly elevated under artemisinin treatment) and glycerophosphodiester diesterase (PF3D7_1406300) (upregulated in artemisinin treatment and higher protein abundance in female gametocytes) (49–51). A conserved *Plasmodium* protein of unknown function recently reported to be downregulated and strongly associated with artemisinin resistance (PF3D7_1328400) was also among the hits (52). Another conserved *Plasmodium* protein (PF3D7_1239800; Atg11) which has been identified to harbor SNPs associated (albeit non-significantly) with artemisinin resistance was among the top targets (53). Thus, DHA treatment was found to significantly alter the chromatin occupancy of PfHDAC1 (majorly depleting the trophozoite-specific occupancy). Among the newly acquired targets under DHA pressure was an assortment of genes responsive to ART pressure, associated with artemisinin resistance, DNA damage repair, autophagy, and gametocyte-specific expression.

### Dihydroartemisinin treatment inhibits PfHDAC1 activity and its post-translational phosphorylation by PfCKII-α

**Fig 5:**
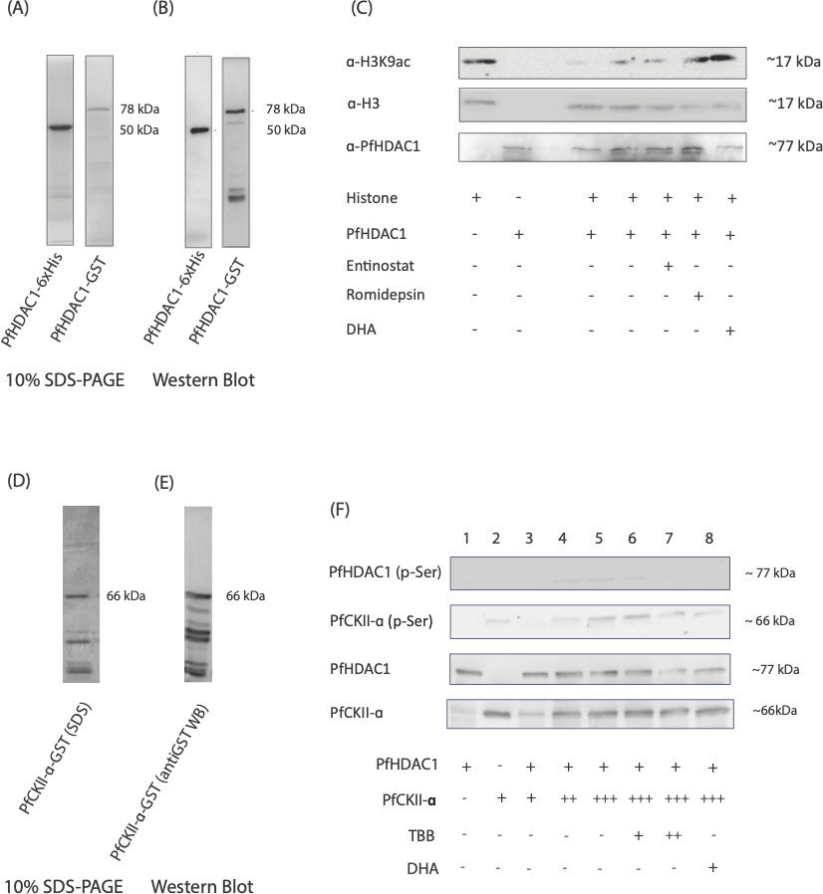
Dihydroartemisinin exposure interferes with *in vitro* catalytic deacetylase activity of PfHDAC1 and its post-translational phosphorylation by PfCKII-α. (**A**) SDS-PAGE (10% resolving) and (**B**) western blotting for purified recombinant PfHDAC1-GST and PfHDAC1-6×His. (**C**) Western blot for the histone deacetylase activity assay probed with α-H3K9ac (top panel), α-H3 (middle panel; loading control), and α-HDAC1 (bottom panel). The reaction components are specified in the following table. Histone H3 was used as the loading control for H3K9ac signal. The inhibitors/antimalarials were used at the following conc.: entinostat (20 µM), romidepsin (360 nM), and DHA (100 nM). The experiments were performed in two biological replicates. (**D**) SDS-PAGE and (**E**) α-GST western blotting for purified recombinant PfCKII-α-GST. (**F**) Western blot (using α-phospho Ser/Thr antibody in top two panels and α-GST antibody in bottom two panels) for *in vitro* kinase activity assay using purified recombinant PfHDAC1 and PfCKII-α. The reaction mix components are as specified in the following table. The conc. of CKII-α inhibitor used was 6 µM (+) and 12 µM (++). The concentration of dihydroartemisinin was 100 nM. Experiments were performed in two biological replicates.

The genomic function of PfHDAC1 is tied-in with its catalytic deacetylase activity and protein-protein interaction-based genomic recruitment. We first investigated if DHA treatment could affect PfHDAC1 deacetylase catalytic activity. GST-tagged recombinant PfHDAC1 was cloned, expressed, and purified from *E. coli* expression system (Fig. 5A and B). We performed *in vitro* histone deacetylase assays using *P. falciparum*-isolated histones as substrate and purified recombinant PfHDAC1 as the enzyme (Fig. 5C). Recombinant PfHDAC1 displayed *in vitro* histone deacetylase activity (observed as depletion of H3K9ac substrate histone modification in western blots). Reaction incubated with 100 nM dihydro-artemisinin showed strong inhibition of the deacetylase activity (Fig. 5C, last lane), confirming that the drug could interfere with the catalytic functions of PfHDAC1. As controls, we used two class I-specific histone deacetylase inhibitors entinostat (MS-275, 20 µM) and romidepsin (360 nM). Both compounds were found to suppress the deacetylase activity associated with PfHDAC1; however, romidepsin exerted a stronger effect than entinostat (Fig. 5C).

The genomic recruitment of HDAC1 (and associated protein-protein interactions) has been linked with its phosphorylation post-translational modification. Mammalian studies strongly indicate that phosphorylation post-translation modification of HDAC1 is mediated by casein kinase (54). A survey of existing blood stage total and phospho-proteome literature indicated that PfHDAC1 is phosphorylated at serine residues, specifically S391, S397, and S440 (55, 56). We analyzed the PfHDAC1 amino-acid sequence on the NetPhos-3.1 web tool, which predicted a strong phosphorylation potential for the S391, S397, and S440 residues and phosphorylation by the CKII kinase at these sites (Fig. S6A through C). The *P. falciparum* ortholog of CKII is PfCKII-α/PF3D7_1108400. A comparison of PfHDAC1 and PfCKII-α gene expression profile showed a robust increase in levels for both genes around early trophozoite stage (~20 HPI) and maintenance of the same into late schizonts (>40 HPI) (41). We sought to test if PfCKII-α could phosphorylate PfHDAC1 and if this phosphorylation could be interrupted by DHA. GST-tagged recombinant PfCKII-α was cloned, expressed, and purified from *E. coli* expression system (Fig. 5D and E). We set up an *in vitro* kinase activity assay where recombinant PfHDAC1 was incubated with increasing amounts of recombinant PfCKII-α. Phosphorylation was detected by resolving reaction mixes on an 10% SDS-PAGE and subsequently probing with an α-phosphoserine/threonine antibody (Fig. 5F; Fig. S6D). A phosphorylated form of PfHDAC1 was observed in reactions with PfCKII-α coincubation with evidence of PfCKII-α auto-phosphorylation activity. Reactions incubated with 100 nM DHA showed inhibition of phosphorylation of PfHDAC1 as well as PfCKII-α autophosphorylation (Fig. 5F, last lane). Control reactions spiked with increasing doses of a PfCKII-α specific inhibitor 4,5,6,7-tetrabromo-2-azabenzimidazole (TBB; 6 and 12 µM) showed inhibition of PfHDAC1 phosphorylation and PfCKII-α auto-phosphorylation (Fig. 5F). These results indicate that DHA can interrupt the deacetylase activity as well as PfCKII-α-mediated phosphorylation of PfHDAC1 in *in vitro* exposure settings, interfering important aspects of its genomic recruitment/function.

## DISCUSSION

In this study, we focused on the class I histone deacetylase, PfHDAC1, which is a prime antimalarial target (21, 22) and is critical for parasite viability (18, 22, 34). We found PfHDAC1 expression and genomic occupancy to be highly dynamic across the stages (ring, trophozoite, and schizont) of the IDC. Ring-stage gene targets were associated primarily with antigenic variation and host cell invasion. The ring-stage expression of host invasion- and antigenic variation-associated genes is likely spillover from merozoite stage (38, 39). Overall, PfHDAC1 occupancy was usually repressive on antigenic variation-associated genes, keeping them suppressed in the ring and trophozoite stages of development. PfHDAC1 occupancy was, however, positively correlated with host cell invasion-associated genes. The enrichment of PfHDAC1 over these genes could govern their timely control as they shut off toward ring-trophozoite transition where they are no longer needed. PfHDAC1 occupancy also positively associated with a few RNA processing and translation-associated genes, likely priming up parasites for trophozoite stage. A major surge in PfHDAC1 gene targets as well as associated pathways was observed in trophozoite stage. Several crucial biological processes, including energy metabolism, proteolysis, DNA replication, chromatin organization, and redox processes, were identified to be targets of PfHDAC1 in this stage. Trophozoite stage is characterized by a strong transcriptional as well as metabolic activation of the parasite, and PfHDAC1 is likely a regulator herein contributing to and tuning robust growth/development of the parasite (11, 39). We found a positive correlation of PfHDAC1 occupancy with the expression of these genes and the downregulation of many of these genes with romidepsin treatment. The wide array of biological functions targeted also reflects the essential nature of PfHDAC1 in the parasite. Toward the end of the IDC in the schizont stage, PfHDAC1 genomic occupancy was found to be reduced and focused mainly on cellular adhesion and ion homeostasis (including Ca^2+^ ion). Ca^2+^ signaling in the parasite is reported to be important for parasite egress and preparing the merozoites for invasion in the subsequent cycle (40). It is highly plausible that the molecular factors that associate with PfHDAC1 change across the IDC stage, thereby governing the balance of timely promoting and shutting down the target genes as and when required.

HDAC1 overexpression has been shown to contribute toward hyper-proliferation phenotypes in mammalian systems by closely regulating the expression of cell cycle factors (57, 58). HDAC1 inhibition in cancer cells is reported to induce cell cycle arrest and suppress invasion and migration (59). Episomal overexpression of PfHDAC1 was found to attribute proliferation advantage to parasites over consecutive invasion cycles. RNA-sequencing of PfHDAC1-GFP-glmS episomal overexpression lines, however, did not reveal changes to genes related with cell cycle or DNA replication but showed strong upregulation of several gene families associated with host cell invasion/egress (merozoite surface, rhoptry structure, etc.) as well as several kinases. We observed increased invasiveness in merozoites from PfHDAC1-GFP-glmS line compared to NF54 control. Through better expression of invasion/egress-associated gene products, PfHDAC1 overexpression may account for invasion and proliferative advantage over infection cycles (60). Among the genes upregulated were those encoding for 6-cystein proteins (P12, P38, and P41) involved in merozoite invasion and merozoite surface proteins (MSP1/2/3/6/7/9/10/11) (61). A number of the latter proteins form the MSP1 complex which serves as a platform for initial attachment with the RBC (42). Additionally, we found upregulation of cytoadherence-linked asexual genes (clag2/3.1/3.2/8/9) and high molecular weight rhoptry genes (RhopH2 and RhopH3) (62). Collectively, these genes encode for the subunits of the *Plasmodium* surface anion channel, a structure responsible for merozoite adhesion to RBC and nutrient acquisition in parasites (63, 64). Genes encoding the rhoptry neck protein (RON2/3/4/5/6/11) which are secreted into the host cell during invasion and contribute to the formation of moving/tight junction were also upregulated (65, 66). Notably, the genes associated with sialic acid-dependent (erythrocyte binding-like ligands) and -independent (reticulocyte homolog) pathway were not enriched for among our upregulated gene set (67). Hence, the effects of PfHDAC1 overexpression may be predominantly associated with enhanced expression of initial attachment and tight-junctions-associated invasion genes and decoupled from influencing expression of cell cycle regulators in *Plasmodium falciparum*. Conversely, using class-specific HDAC inhibitors, we observed a deceleration of parasite progression through the IDC. This was also found to correlate with distortion in proper morphological development (especially DNA replication) and carry over of the infection to the next cycle. Using RNA-sequencing (in trophozoite stage), we identified the effects of romidepsin treatment on parasites and manifested in the form of deregulation of key genes associated with a wide array of biological processes, including RNA splicing, translation, transcriptional regulation, cell cycle, DNA replication, and parasite interaction with host cell (entry/egress). Improper DNA replication caused by downregulation of associated genes may be responsible for insufficient conversion of “DNA light” forms of parasites (rings and early trophozoites) into “DNA heavy” forms resulting in faulty maturation.

Interestingly, PfHDAC1 overexpression was found to establish artemisinin resistance in sensitive parasites and increase the resistance level in Kelch13 mutant parasites. Previous studies have reported pharmacological suppression of PfHDAC1 to be effective in eliminating dihydroartemisinin-resistant parasites (34). Our findings are possibly a reflection of the same. We found suppression of PfHDAC1 protein levels upon DHA treatment, and it is possible that, in PfHDAC1 overexpression parasites, the additional abundance of PfHDAC1 could possibly counteract the effects of the drug, providing a degree of resistance. Scoping the transcriptome of PfHDAC1 overexpression parasites, we note the downregulation of several mitochondrial electron transport chain factors. Mitochondrial biology has remained a subject of much speculation in terms of role in activation of artemisinin and contribution toward artemisinin resistance (10, 68, 69). Future experiments shall be focused on dissecting the role of PfHDAC1 overexpression in artemisinin resistance.

Furthermore, DHA treatment in trophozoites was found to alter the genomic occupancy of PfHDAC1, leading to its eviction or depletion from trophozoite target sites. Closer inspection of the newly acquired PfHDAC1 targets under DHA pressure revealed genes associated with DNA damage repair and autophagy (PF3D7_1031300 and PF3D7_1234300). DHA has been reported to form adducts and is considered to evoke DNA damage (70). Furthermore, we observed enrichment of PfHDAC1 over genes that have been reported to be deregulated (transcript/protein) in artemisinin-treated parasites (PF3D7_1406300 and PF3D7_1361600). Two ApiAP2 factors (PF3D7_1222400 and PF3D7_0604100) reported to be strongly expressed in AP2G^+^- and LysoPC-depleted (gametocyte) cells were also under PfHDAC1 occupancy in DHA stress (48). Interestingly, induction of gametocytogenesis has been reported as a potential flight mechanism in parasites exposed to artemisinin (71). Finally, PF3D7_1328400 (downregulation associated with artemisinin resistance in transcriptome-wide association studies) and Atg11/PF3D71239800 (SNPs associated with artemisinin resistance) were also among top PfHDAC1 occupancy hits (52, 53). Thus, PfHDAC1 could possibly respond to stress evoked by DHA and acquire new targets contributing to the drug-related phenotype.

We finally identified that DHA treatment inhibited *in vitro* histone deacetylase activity of PfHDAC1. Furthermore, PfHDAC1 underwent *in vitro* PfCKII-α-mediated phosphorylation modification, which was interrupted by DHA exposure. This highlights crucial aspects of PfHDAC1 functionality (especially genomic recruitment/function), which are sensitive to drug exposure. Previously, a cyclin-dependent kinase 2-related kinase 3 (Pfcrk3) has been shown to be associated with complexes that additionally possess histone deacetylase activity (72). Which exact histone deacetylase this kinase was associated with has not been identified, but it leads credence to interaction of kinases with HDACs in *Plasmodium falciparum*. The phosphorylation PTM adds an important layer toward the regulation of PfHDAC1 function. PTMs are known to modulate protein-protein interactions for HDACs, which in context of PfHDAC1 could be important for regulating its genomic recruitment by interaction with transcription factors (54). Interestingly, PfCKII has been previously identified to phosphorylate members of invasion family proteins, including erythrocyte binding-like antigens and reticulocyte binding-like protein homologs, and this modification is likely to be important for efficient invasion of host RBCs (73).

Overall, out study highlights the genetic targets under PfHDAC1 epigenetic regulation in the parasite and its essential role in governing asexual parasite host cell invasion and viability. We additionally identify PfHDAC1 to be an epigenetic response factor to dihydroartemisinin stress in parasites with its higher abundance associated with resistance to the drug.

## MATERIALS AND METHODS

### Generation of GFP knockin constructs for PfHDAC1

For the GFP knockin construct, 750 bp of the C-terminal region of PfHDAC1 was cloned into the parent pSLI-2xFKBP-GFP plasmid (originally generated by Dr. Tobias Spielmann team) (37).

### *P. falciparum* transfections

Lonza nucleofector 4D was used for transfection of plasmids into highly synchronized parasites. Eighty micrograms of knock-in plasmid was dissolved in 100 µL of P3 primary solution. Double synchronized (Percoll centrifugation) segmented schizonts were mixed with the DNA/P3 solution mix and nucleoporated in the nucleofector 4D machine with the pulse program FP 158 (designed for *P. berghei*). The zapped cells were immediately transferred to a T25 flask with 3 mL of media and 200 µL fresh RBC and set on a shaker incubator at 37°C for 2 hours to allow the egressed merozoites to invade the fresh cells. The flask was later supplemented with additional media to make it to 2% hematocrit. Drug selection was started 24 hours post transfection. Appearance of transgenic lines was checked initially via Giemsa smear.

### Cloning of the transgenic parasite lines

The validated transgenic lines were serially diluted in a 96-well flat plate to obtain 200 µL cultures with finally one parasite per well. These were confirmed with plaque formations and then transferred onto round bottom 96-well plates for expansion. The cells were then microscopically confirmed for GFP expression and then expanded into flasks.

### Validation of PfHDAC1-2×FKBP-GFP transgenic line

Confocal microscopy and western blotting using anti-GFP primary antibody (abcam, ab290; 1:2,000 dilution in 5% milk/TBST) were used to validate the PfHDAC1-2×FKBP-GFP transgenic line generated from NF54 parasites. NF54 wild type and uninfected RBC lysate were used as controls in western blotting. α-Actin (Sigma A2066; 1:2,000 dilution) probing was done for loading control. The transgenics were generated at Dr. Moritz Treeck’s lab at The Francis Crick Institute, London, UK. Further validation of the knockin line was performed using integration-specific PCR amplification. A combination of primers targeting different loci was used for this purpose, as follows: primer P1, upstream of the homology region 1; primer P2, within 2×FKBP sequence; primer P3, within GFP sequence; primer P4, within 3′ UTR of PfHDAC1. For sequence-level validation, we used the whole-genome sequencing data of the pre-chromatin immunoprecipitation input. The sequence was mapped against a modified FASTA file containing the PfHDAC1 coding sequence fused with 2×FKBP-GFP modification at the 3′ end. The alignment BAM file was visualized on IGV browser.

### Immunofluorescence assay for PfHDAC1-2×FKBP-GFP knockin parasites

A 2 mL culture of stage-specific knockin parasites was washed using 1× PBS thrice and fixed with 4% paraformaldehyde with 0.075% glutaraldehyde solution for 30 minutes at 37°C. The samples were permeabilized using 0.1% Triton X-100 solution for 10 minutes at room temperature. The pellet was blocked with 5% bovine serum albumin for 1 hour at room temperature. The primary antibody anti-GFP (CST antibodies #2956) was added at 1:500 dilution and incubated overnight at 4°C and 8 rpm. The pellet was washed five times using 1× PBS and incubated with anti-rabbit Alexa Fluor 488 secondary antibody (1:2,000 dilution) for 1 hour at room temperature in dark condition. The pellet was washed five times with 1× PBS and prepped into smears. The smear was fixed using methanol, and ProLong Gold antifade with DAPI (Invitrogen catalog: P36941) was used for mounting the slides. The imaging was done on EVOS M7000 Fluorescent microscope and analyzed on ImageJ software.

### Chromatin immunoprecipitation

PfHDAC1-2×FKBP-GFP knock-in NF54 line parasites were tightly synchronized with 2× rounds of Percoll density gradient centrifugation and cultured at 5% parasitemia and 2% hematocrit. Samples were harvested at 10 HPI (ring), 21 HPI (early trophozoite), 24 HPI (trophozoite), and 40 HPI (schizont). For DHA treatment ChIP, tightly synchronized parasites were treated with 100 nM DHA (for 3 hours; 21–24 HPI) and then harvested. The cultures were crosslinked using 1% formaldehyde (Thermo Scientific, 28908) for 10 minutes at RT. Glycine (150 mM) was added for quenching the cross-linking reaction. The samples were washed using 1× PBS (chilled) before proceeding with lysis. Sample homogenization was performed using swelling buffer (25 mM Tris-Cl pH 7.9, 1.5 mM MgCl_2_, 10 mM KCL, 0.1% NP-40, 1 mM DTT, 0.5 mM PMSF, 1× PIC) followed by cell lysis in sonication buffer (10 mM Tris-Cl pH 7.5, 200 mM NaCl, 1% SDS, 4% NP-40, 1 mM PMSF, 1× PIC). Sonication was performed using Covaris S220 to obtain the chromatin size of 200–400 bp. Pre-clearing was performed for 1 hour at 4℃ using recombinant protein A conjugated Dynabeads (Invitrogen, 1001D), beads with continuous gentle inverting. Purified chromatin (30 µg) was used per immunoprecipitation reaction. α-GFP antibody (2 μg; abcam, ab290) was incubated with the chromatin for 12 hours at 4°C. Samples were then incubated with saturated protein A Dynabeads beads for 4 hours at 4°C. Bound chromatin was finally washed with low salt, high salt, and LiCl wash buffers followed by TE buffer wash and eluted using ChIP elution buffer (1% SDS, 0.1 M sodium bicarbonate). DNA from pre-IP-sonicated chromatin was taken as input for downstream normalization. Both the ChIP and input samples were reverse cross-linked using 0.3 M NaCl overnight at 65°C along with RNase A treatment. Proteinase K treatment was performed at 42°C next day for 1 hour. Finally, DNA was purified using phenol:chloroform:isoamyl alcohol precipitation.

### ChIP-sequencing library preparation and sequencing

ChIP-sequencing libraries were prepared from 5 to 10 ng of DNA using the NEBNext Ultra II DNA Library Prep kit. Chromatin-immunoprecipitated, fragmented DNA samples were end repaired and adapters ligated. Size selection was performed using Agencourt XP beads (Beckman Coulter). Adapter-ligated fragments were PCR amplified using indexing primers followed by purification using the Agencourt XP beads (Beckman Coulter). The library electropherograms were assessed using Agilent Bioanalyzer 2100 and Agilent DNA 1000 Kit. The sequencing for these was done at the next-generation sequencing facility at IISER, Pune using the Illumina NextSeq 550 sequencer using 1 × 75 bp run specs.

### ChIP-seq data analysis

The sequencing reads were demultiplexed using the bcl2fastq tool in the Linux platform. Read quality check was performed using FASTQC, and subquality reads (not passing Q30 filter) and adaptor sequences were trimmed using Trim_Glaore. Reads were aligned onto the *Plasmodium falciparum* 3D7 reference genome ver. 53 using Bowtie aligner. SAMTools was used to sort the data files. The sorted ChIP and input BAM files were then used for peak calling in MACS2 software (broad peak, no model). Background subtraction was performed using the bdgcmp command in MACS. We considered peaks having ≥2-fold enrichment to be suitable candidates as target genes. Peak annotation was performed using Bedtools using a modified GTF file (including additional 1 kb upstream region of genes as promoter), and the peaks were visualized using IGV genome browser (74, 75).

Deeptools suite was used for additional ChIP data analysis and visualization (76). bamCompare command was used to generate the log2 ChIP/input normalized bigwig files (with a prior RPKM normalization of both ChIP and input bam files). multiBigwigsummary command was used to compare the log2ChIP/input bigwigs of all the stage data sets using a modified GTF file as a reference (including additional −1 kb upstream region as gene promoter). PlasmoDB gene ontology tool was used to identify the biological pathways enriched for PfHDAC1 occupancy gene targets (77). Custom script was to generate the GO dotplots based on PlasmoDB output.

For ChIP occupancy and gene expression correlation, the expression levels of target genes in each stage were collected from published RNA sequencing data set (roughly 8-hour interval across IDC) (41). Expression matrix of target genes was plotted using Morpheus heatmap online tool and in-build hierarchical clustering applied to identify clusters of gene expression pattern for the targets.

### Generation of overexpression constructs for PfHDAC1

The full-length sequence of PfHDAC1 was PCR amplified from genomic DNA using sequence specific primers and cloned into the pDC2 overexpression vector using the AvrII and NheI restriction sites. This put the PfHDAC1 under control of the calmodulin promoter and in frame with a C-terminal GFP tag. Furthermore, to add a layer of regulatability to the overexpression system, we cloned a glmS ribozyme sequence (PCR amplified from pHSP101 plasmid) in frame with the GFP tag using XhoI enzyme. This resulted in an overexpression system synthesizing PfHDAC1-GFP -glmS fusion transcript.

### *P. falciparum* transfections for PfHDAC1-GFP-glmS overexpression constructs

Transfections for control (GFP-glmS) and test (PfHDAC1-GFP-glmS) were performed in artemisinin-sensitive PfKelch13WT (MRA-1252 parasite line sourced from the MR4 repository) and artemisinin-resistant PfKelch13 R539T mutant (MRA-1240) (47). The transfection protocol was followed as described previously, except that 30 ug of overexpression plasmid was used. Drug selection was started 24 hours post transfection with 2 µg/mL Blasticidin-S. Confirmation of GFP expression and clonal selection of transgenic parasites were done as described previously for the PfHDAC1-2×FKBP-GFP knockin line. The transgenics were generated and tested upon at Dr. Moritz Treeck’s lab at The Francis Crick Institute, London.

### Validation of reversible overexpression of PfHDAC1-GFP

Validation of PfHDAC1-GFP overexpression and reversible depletion was validated with treatment of culture with 0, 2.5, 5, and 7.5 mM of glucosamine-HCl followed by western blotting for depletion of GFP-tagged PfHDAC1 signal.

### Parasite growth curve assay (flow cytometry at Crick Institute, London)

The GFP-glmS ctrl overexpression and PfHDAC1-GFP-glmS overexpression were tightly synchronized using two rounds of Percoll density gradient centrifugation. Parasitemia was estimated by staining cells with SYBR Green I dye and subjecting them to flow cytometry. The cultures were diluted to 1% starting parasitemia and 2% hematocrit in 96-well plates and allowed to proliferate over a duration of 6 days/3 IDC. For testing the effect of glucosamine dosage (and episomal expression suppression) on the growth trend, wells with 2.5 mM glucosamine treatment were set up in parallel with mock-treated parasites for both PfHDAC1-GFP-glmS overexpression and GFP-glmS overexpression lines. Media were replenished carefully after the third day with accurate replacement for mock and drugs. Each strain and condition were set in triplicate on the 96-well plate. The culture in the wells was sampled off every 24 hours until the finish of the assay and subjected to SYBR Green I flow cytometry to calculate parasitemia progression over the course of the experiment. Growth curves were plotted on GraphPad Prism software.

### PfHDAC1 overexpression parasite growth assay by flowcytometry (IISER Pune)

Tightly synchronized cultures of PfHDAC1-GFP-glmS overexpression and control GFP-glmS overexpression were adjusted to 2% parasitemia and 2% hematocrit. An uninfected RBC culture at 2% hematocrit was used as background control. At 10 HPI (in midring stage), 1 mL culture volume from each culture setup was subjected to staining with 1× DAPI for 30 minutes at room temperature in dark. After washing off excess stain, individual samples were passed through BD FACS system and recorded for at least 1,00,000 events. Stained uninfected RBCs were used for gating background signal for DAPI. The cultures were subsequently tested at 3 and 5 days post invasion. All analysis was done using BD FACSDiva 8.0.1.1 software. Additionally, tightly synchronized (3 hour invasion window) cultures of PfHDAC1-GFP-glmS parasites were cultured at 4% parasitemia and 2% hematocrit under increasing dosage of glucosamine-HCl (0, 2.5, 5, and 7.5 mM) and sampled for flowcytometry-based counting every 48 hours (in midtrophozoite stage) for three cycles of growth. Two-way ANOVA multiple testing was performed to compare parasitemia across different treatments at each sampling point. Furthermore, 10 µM E64 (trans-epoxysuccinyl-L-leucylamido(4-guanidino) butane, Sigma product number E3132) was added to the culture after appearance of initially segmented schizonts in the third cycle to arrest merozoite egress from mature schizonts. Merozoite counting was performed from Giemsa smears in each treatment culture. An unpaired *t*-test was used to perform statistical comparison of counts across treatment.

### Intraerythrocytic development and merozoite counting for PfHDAC1 and GFP overexpression parasites

Tightly synchronized PfHDAC1-GFP-glmS and GFP-glmS overexpression lines were followed every 8 hours (from 10 to 4 HPI of second invasion cycle). Culture samples were taken for Giemsa microscopy, and the parasites from each stage were counted and inspected for any shift in stage. Additionally, from a parallel culture, PfHDAC1 overexpression and control GFP overexpression lines were subjected to schizont enrichment using 63% Percoll gradient. Collected parasites were fixed on slide using methanol and stained with 10% Giemsa solution. For each condition, segmented merozoites present per schizonts were counted manually. Thirty schizonts per condition were analyzed.

### Culture for RNA sequencing of PfHDAC1 overexpression and PfHDAC1 pharmacological inhibition

For strand-specific RNA sequencing, parasites carrying the control GFP-glmS episomal construct and PfHDAC1-GFP-glmS episomal construct were cultured in triplicate at 8% parasitemia and 2% hematocrit. Post double synchronization, the cultures were harvested at roughly 24 hours post invasion timepoint. For strand-specific RNA sequencing upon mock vs romidepsin treatment, NF54 strain parasites were cultured in triplicates at 8% parasitemia and 2% hematocrit. Post double synchronization, one set of replicates was treated with DMSO mock, while the test set of replicates was treated with 360 nM romidepsin (2× IC_50_) for 3 hours at 21 HPI and harvested at 24 hours post invasion.

### RNA isolation and RNA sequencing library preparation

Post harvesting the parasites, we proceeded with isolation of RNA from the TriZol-suspended samples. The Thermo Colibri 3′ mRNA library preparation kit was utilized for preparation of strand-specific libraries. The final amplified libraries were quality checked on Agilent bioanalyzer for size distribution and Qubit fluorometer for quantity. Single-end sequencing was done on the Illumina NextSeq 550 platform (150 bp read length).

### RNA sequencing and data analysis

The sequencing data were quality checked using FASTQC and trimmed for low-quality (Q30) and adaptor sequences. The trimmed reads were then mapped onto *Plasmodium falciparum* 3D7 genome ver. 53 using Hisat2 (unpaired reads and reverse-stranded orientation settings). HTSeq-count was used to quantify the raw reads from the mapped data sets, and then DESeq2 was used for sample-wise read normalization and differential gene expression analysis with the cognate test vs ctrl samples (PfHDAC1 overexpression vs GFP overexpression; romidepsin vs mock treatment). Figures and plots were generated with the R environment and GraphPad Prism software. Additionally, RNA sequencing BAM alignments from PfHDAC1-GFP-glmS and GFP-glmS overexpression lines (in both artemisinin-sensitive PfKelch13 wild-type background and -resistant PfKelch13R539T mutant background) were visualized on IGV genome browser to confirm the status of mutations in the parental parasite lines.

### Merozoite invasion assay

Tightly synchronized cultures (3 hours invasion window) of NF54 and PfHDAC1-GFP-glmS overexpression lines were maintained at 5% parasitemia and 3% hematocrit. Mature segmented schizonts were purified and isolated using Percoll density gradient and resuspended in 1 mL of 1× PBS solution. The schizonts were passed through a sterile 3 µm filter to isolate the merozoites which were then counted using an improved Neubauer’s chamber. The merozoite suspension was mixed with 1% fresh RBCs (10 µL) and incubated on an orbital shaker at 37°C for 30 minutes to allow reinvasion. The RBCs were pelleted down and supplemented with complete RPMI media and seeded onto a 96-well plate in triplicates. After 16 hours of incubation, the culture was stained with 1× DAPI and counted using FACS (BD Aria). Simultaneously, the parasites were observed microscopically using smears. The invasion rate was calculated as follows: invasion rate = (% parasitemia) × (no. of erythrocytes per microliter/no. of merozoites per microliter).

The invasion rate of PfHDAC1-GFP-glmS overexpression line was normalized with that of NF54 control and plotted using GraphPad prism 8. The statistical analysis was done using an unpaired *t*-test.

### Investigation of the effect of PfHDAC1 inhibition upon intraerythrocytic developmental cycle

Highly synchronized (Percoll density gradient centrifugation) parasites were cultured at ~5% parasitemia and 2% hematocrit and treated with 0.5× IC_50_ concentration of PfHDAC1 inhibitor romidepsin at roughly 6 hours post invasion (regarded as 0 hours post treatment). We had calculated the IC_50_ of romidepsin at 180 nM previously. The parasites were then sampled off at every 8 hours for the next 48 hours and frozen for either SYBR fluorometry to estimate the DNA content or prepared into thin Giemsa-stained smears for microscopic examination of morphology and stage progression. Relative percentage of parasites from each of the developmental stages of *P. falciparum* was calculated and plotted on histograms. Each timepoint is validated with at least 1,000 cell count observations. The SYBR fluorometry samples were lysed with the SYBR Green I lysis buffer and processed for DNA signal intensity via fluorometry (excitation: 495 nm; emission: 520 nm). The relative DNA signal for inhibitor treatment estimated by fluorescence was plotted as a percentage against mock sample readouts. For parasite proliferation estimation, parasitemia was recounted upon completion of one cycle in the mock vs inhibitor-treated samples.

### DHA- and PfHDAC1-specific inhibitor treatment for protein/histone modification analysis

Parasite culture grown at 5% parasitemia and 2% hematocrit was treated with DMSO mock or 2× IC_50_ dosage of entinostat or romidepsin for 8 hours (20–28 HPI; early trophozoite stage) prior to harvesting for estimation of effect on protein levels with western blotting (α-GFP/HDAC1; α-BiP and α-HSP70). Additionally, treatment with 50 nM DHA was performed and processed for western blotting (α-GFP/HDAC1; α-BiP and α-HSP70). Parasite cultures grown at 5% parasitemia and 2% hematocrit were treated with DMSO mock or 2× IC_50_ dosage (360 nM) of romidepsin for 3 hours (21–24 HPI; early trophozoite stage) prior to harvesting for estimation of effect on H3K9ac histone modification levels with western blotting (α-H3K9ac Millipore 06-942 ab) used at 1:2,000 dilution. α-Actin antibody (Sigma A2066) was used as loading control (1:2,000 dilution).

### Ring-stage survival assay

The ring-stage survival assay was performed on PfKelch13 WT and mutant R539T parasites (MRA-1252 and MRA-1240) which were transfected for overexpression of PfHDAC1-GFP-glmS or GFP-glmS control construct. Tightly synchronized (double Percoll density gradient centrifugation) 0–3-hour *P. falciparum* ring-stage parasites were taken at 1.5% parasitemia and 2% hematocrit in 200 µL volume 96-well plates. Parasites were then either treated with DMSO mock or with 700 nM dihydroartemisinin for a duration of 6 hours, following which the mock/drug was washed off, and the parasites were reinstated in culture. They were allowed to grow for another 66 hours. At the end of 72 hours, the live parasitemia was calculated by flow cytometry for SYBR Green I and Mito Tracker signal in the DHA- vs mock-treated culture. The percentage of cells surviving DHA treatment relative to the mock treatment was logged as %RSA. We calculated the %RSA for one clone each of GFP-glmS overexpression parasites (Kelch13 WT and mutant). Two independent clones of PfHDAC1 overexpression were tested for RSA in PfKelch13 mutant background, while one PfHDAC1 overexpression clone was tested in PfKelch13 WT background. %RSA were compared across the appropriate lines. While the parasite lines were maintained under BSD selection to select for the episomal OE plasmid, the drug pressure was taken off at the start of RSA to avoid any potential cross interference of DHA with BSD.

### Cloning, overexpression, and purification of recombinant PfHDAC1 and PfCKII-α

The full-length PfHDAC1 sequence was PCR amplified and cloned into the pET28a+ bacterial expression vector (NheI and XhoI restriction enzyme sites) and into the pGEX-4T1 expression vector (BamHI and XhoI sites) to get a 6×His-tagged and GST-tagged version of the gene, respectively. The overexpression constructs were transformed independently into the BL21 (DE3) *E. coli* expression system. Bacterial culture growing at 0.5 optical density at 600 nm was induced with 0.5 mM isopropyl-1-thio-β-d-galactopyranoside IPTG for 5 hours at 25℃. The bacterial pellet was resuspended in sonication buffer (10 mM Tris-Cl pH 8.0, 150 mM NaCl, 10% glycerol, 1× PIC, and 1× PMSF) followed by sonication using probe sonicator (Thomas Scientific, 70% amplitude, 10 minutes, 02 s on/ 06 s off). After the sonication lysate was centrifuged at high speed 14,000 RPM for 30 minutes at 4°C, the supernatant was stored at −80℃, and the pellet was resuspended in 8 M Urea prepared in the sonication buffer. Pellet was kept in shaking condition for 1 hour at room temperature followed by centrifugation. The earlier supernatant as well as pellet supernatant was run on 10% resolving SDS PAGE gel along with uninduced bacterial lysate to check the induction of protein expression. Protein expression was also confirmed using western blotting (anti-His or anti-GST antibody). Once the expression was confirmed, protein purification was performed. The GST-tagged PfHDAC1 was purified from the soluble fraction with glutathione sepharose 4B beads (GE Healthcare) using 20 mM reduced glutathione. The 6×His-tagged protein was purified from the insoluble fraction of a separately induced culture using Ni-NTA beads (GE Healthcare) with 250 mM imidazole. Purification of proteins was checked using SDS PAGE and western blotting. Purified proteins were dialyzed and stored at −20°C.

The PfCKII-α was PCR amplified from genomic DNA and cloned into the pEX4T1 vector for bacterial expression. GST-tagged PfCKII-α clone was transformed into BL21 DE3 *E. coli*-competent cells. Protein induction was performed with 1M IPTG (at OD 0.6), and protein expression was allowed for 12 hours at 18°C. Cell lysate was prepared as described for PfHDAC1-GST tagged above, and purification of protein was done using glutathione beads. The purified protein was eluted with 10 mM reduced glutathione and dialyzed subsequently.

### Antibodies

α-Actin (Sigma A2066) and α-Rabbit IgG (OSB PM035) were used for western blotting and immunoprecipitation, respectively. Goat α-Rabbit Alexa Fluor 647 (Invitrogen A21245), Goat α-Rat Alexa Fluor 488 (Invitrogen A11006), and Goat α-Rabbit Alexa Fluor 488 (Invitrogen A11034) were used for immunofluorescence. Additionally, α-H3K9ac (Millipore 06-942), α-his (Biobharati Life Sciences BB-AB0010), α-GST (Cloud Clone Corp.), and α-phosphoserine/threonine (Abcam ab17464) were also used for western blotting. Rabbit polyclonal antibodies against PfHDAC1 were generated by immunizing rabbits with purified his-tagged recombinant PfHDAC1 (conjugated to Freund’s incomplete and complete adjuvant). The New Zealand White rabbits (3–4 months old) were used for antibody generation. Furthermore, 200 µg protein was intramuscularly injected for the first round followed by four booster doses of 150 µg each. Five bleeds were collected via femoral bleeds and centrifuged for obtaining the antisera from whole blood. The antibodies were affinity purified using the GST-tagged recombinant PfHDAC1 conjugated to SulfoLink resin (Thermo catalog 20402).

### *In vitro* deacetylase activity assay

Highly purified recombinant PfHDAC1 was used as the enzyme, and histones isolated from the *P. falciparum* pellet were used as substrate for this reaction. Two micrograms of histones was incubated with 2 µg of PfHDAC1 (procured after the kinase activity assay) in the presence of a deacetylase buffer (25 mM Tris HCl, pH 8.0, 137 mM NaCl, 2.7 mM KCl, 1 mM MgCl_2_, 0.1 mM ZnCl_2_). For control reaction, only purified histones were incubated in the deacetylase reaction mix (without PfHDAC1). Reaction with 100 nM dihydroartemisinin was also set up to investigate the effect of the antimalarial on the catalytic activity. Class I HDAC-specific inhibitors romidepsin (360 nM) and entinostat (20 µM) were used in additional reactions to inhibit the potential deacetylase activity of PfHDAC1. Post incubation at 37°C for 1 hour, the reactions were run on a 15% resolving SDS-PAGE, and western blotting was done using α-H3K9ac antibody. The blot was subsequently stripped and probed with α-H3 antibody (substrate loading control) and α-HDAC1 antibody (enzyme loading control).

### *In vitro* kinase activity assay

Highly purified recombinant PfHDAC1 and PfCKII-α were used as substrate and enzyme for *in vitro* kinase assay. And, 500 ng of PfHDAC1 was incubated with increasing amounts (360, 780, and 1,080 ng) of PfCKII-α in the presence of a kinase activity buffer (20 mM Tris HCl pH 7.5, 20 mM MgCl_2_, 2 mM MnCl_2_,0.1 mM PMSF) with 10 µM ATP. For a baseline control, phospho-PfHDAC1 incubated without PfCKII-α was taken. To probe the effect of artemisinin treatment on the kinase reaction, we set up an additional reaction with 100 nM dihydroartemisinin. Furthermore, two reactions with increasing dosages of a PfCKII-α-specific inhibitor 4,5,6,7-tetrabromobenzotriazole and TBB (Merck/Calbiochem CAS 17374–26-4) were taken to check for possible suppression of the phosphoryl group transfer. The reactions were incubated at 37℃ on a thermal mixer and then run on 10% resolving SDS-PAGE gel followed by western blotting with anti-phospho-serine antibody.

## Data Availability

ChIP-sequencing data for PfHDAC1 as well as gene expression data (RNA sequencing) for different conditions are submitted to Sequence Read Archive (SRA) under BioProject ID PRJNA817874.

## SUPPLEMENTAL MATERIAL

Figure S1 mbio.02377-23-s0001.tiff PfHDAC1-2×FKBP-GFP results.

Figure S2 mbio.02377-23-s0002.tiff PfHDAC1-GFP-glmS results.

Figure S3 mbio.02377-23-s0003.tiff Parasite progression.

Figure S4 mbio.02377-23-s0004.tiff PfHDAC1 results.

Figure S5 mbio.02377-23-s0005.tiff PfKelch13 results.

Figure S6 mbio.02377-23-s0006.tiff PfHDAC1 results.

Supplemental Material and Table 1 mbio.02377-23-s0007.pdf Supplemental text, figure and table legends, and Table S1.

Table S2 mbio.02377-23-s0008.xlsx Details of PfHDAC1 ChIP peaks called by MACS2 software.

Table S3 mbio.02377-23-s0009.xlsx Details of the DESeq2 differential gene expression analysis.

Table S4 mbio.02377-23-s0010.xlsx Log2(fold change) across genes deregulated upon romidepsin treatment.

ASM does not own the copyrights to Supplemental Material that may be linked to, or accessed through, an article. The authors have granted ASM a non-exclusive, world-wide license to publish the Supplemental Material files. Please contact the corresponding author directly for reuse.
