# Supplementary figures and images for: PfHDAC1 is an essential regulator of P. falciparum asexual proliferation and host cell invasion genes with a dynamic genomic occupancy responsive to artemisinin stress
Source: mBio. 2024 May 6;15(6):e02377-23. doi: 10.1128/mbio.02377-23 (PMC11237754; doi:10.1128/mbio.02377-23)

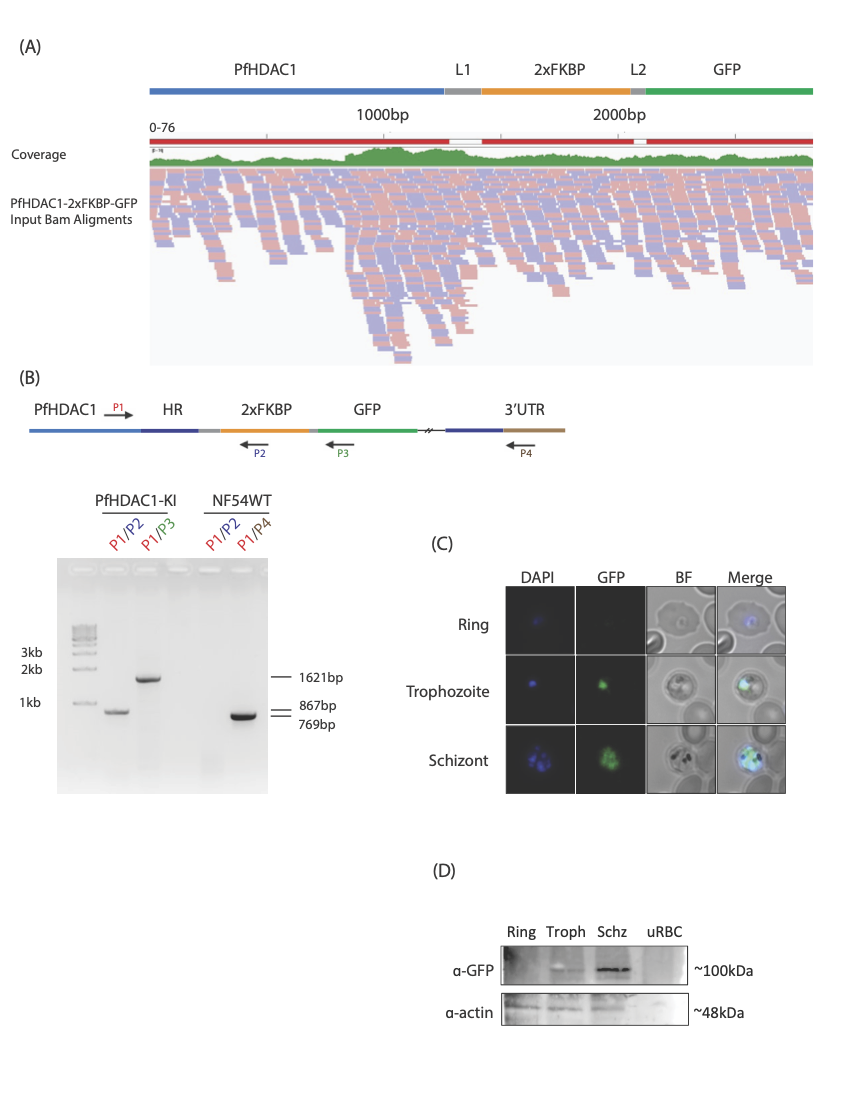

Supplement: Figure S1 — PfHDAC1-2×FKBP-GFP results. [file mbio.02377-23-s0001.tiff]

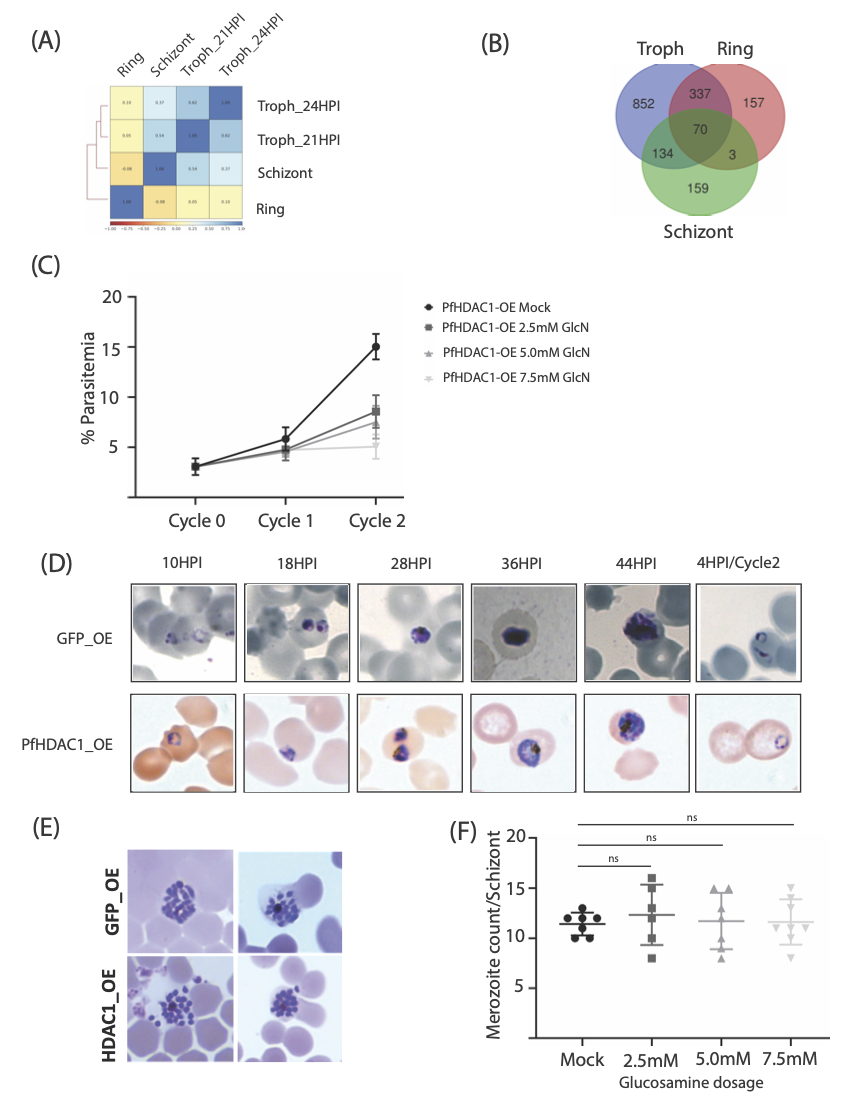

Supplement: Figure S2 — PfHDAC1-GFP-glmS results. [file mbio.02377-23-s0002.tiff]

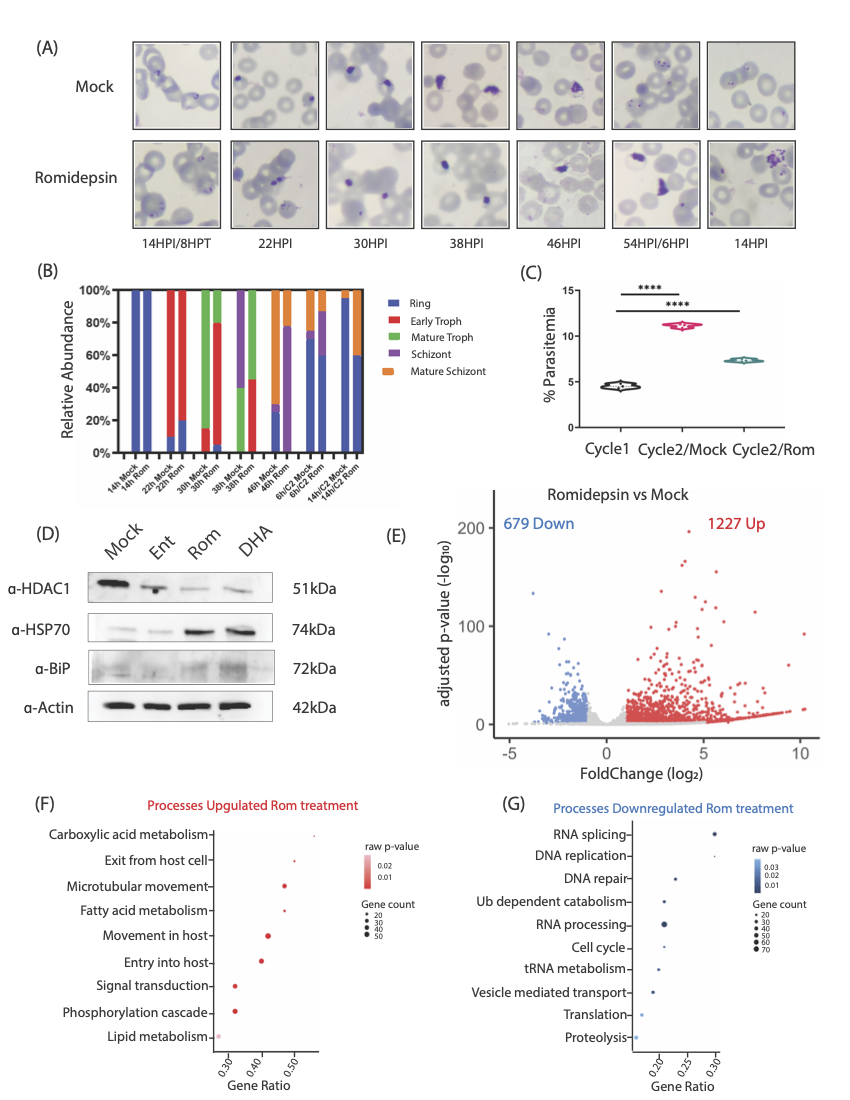

Supplement: Figure S3 — Parasite progression. [file mbio.02377-23-s0003.tiff]

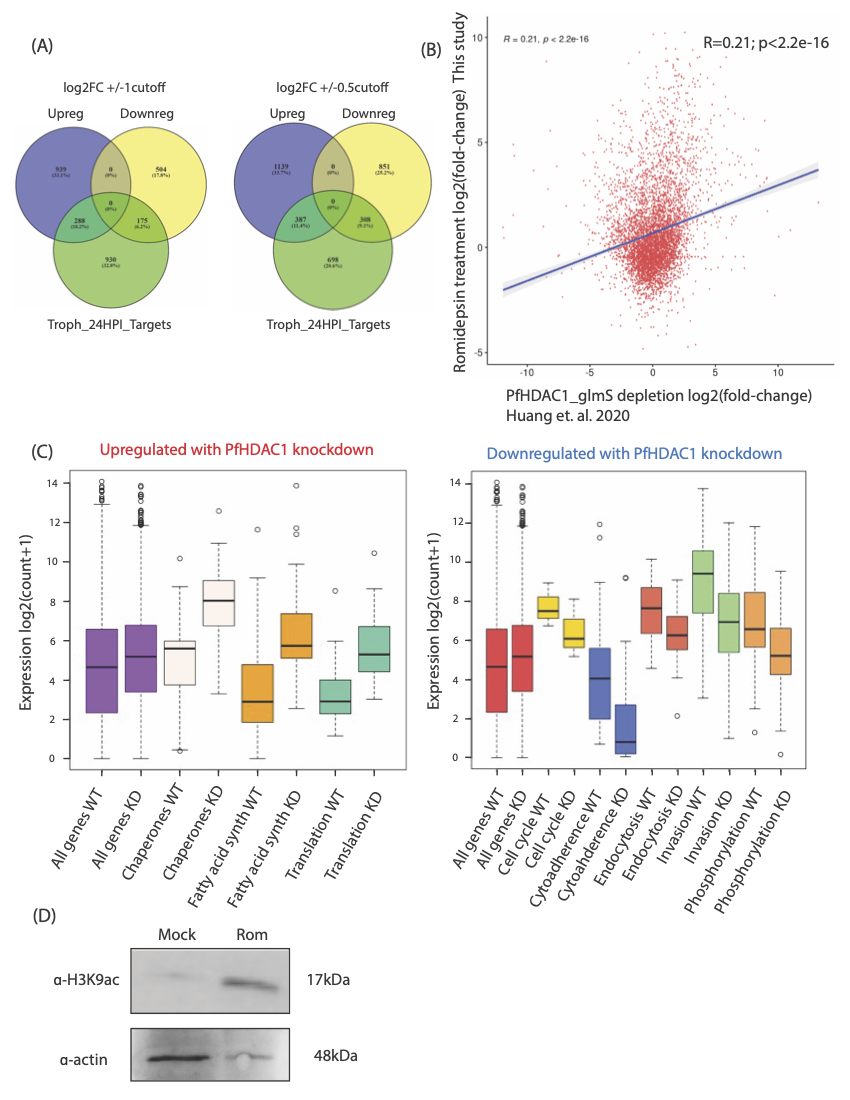

Supplement: Figure S4 — PfHDAC1 results. [file mbio.02377-23-s0004.tiff]

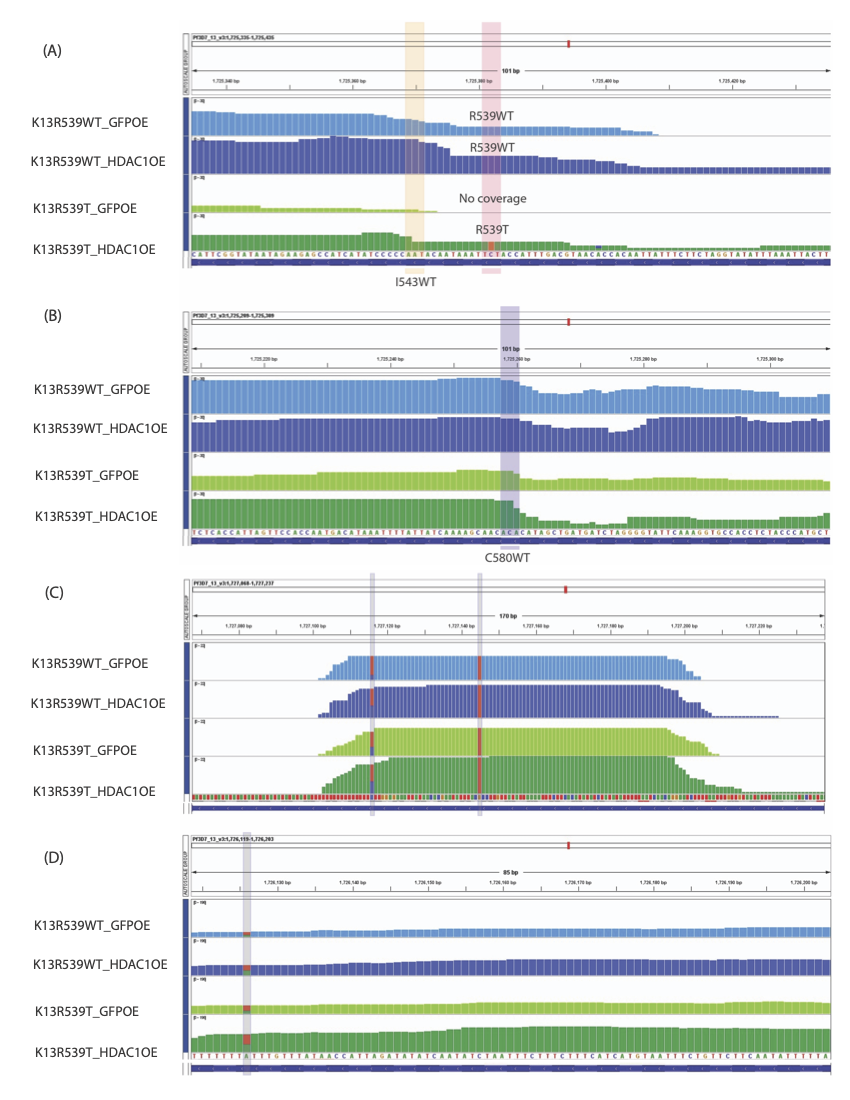

Supplement: Figure S5 — PfKelch13 results. [file mbio.02377-23-s0005.tiff]

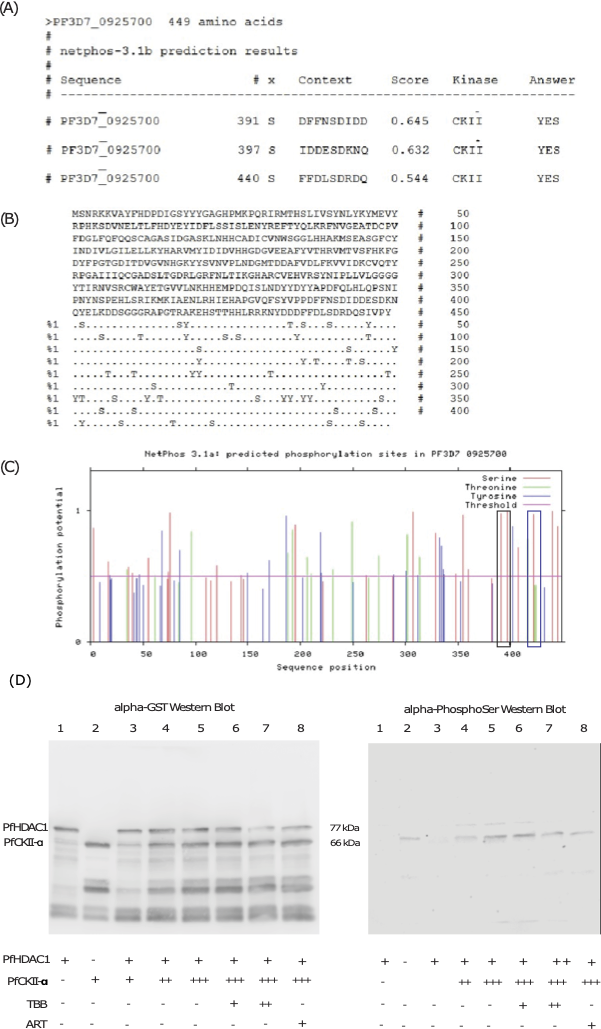

Supplement: Figure S6 — PfHDAC1 results. [file mbio.02377-23-s0006.tiff]
