# Supplementary material for: PfHDAC1 is an essential regulator of P. falciparum asexual proliferation and host cell invasion genes with a dynamic genomic occupancy responsive to artemisinin stress
Source: mBio. 2024 May 6;15(6):e02377-23. doi: 10.1128/mbio.02377-23 (PMC11237754; doi:10.1128/mbio.02377-23)
Supplement: Supplemental Material and Table 1 — Supplemental text, figure and table legends, and Table S1. [file mbio.02377-23-s0007.pdf]

PfHDAC1 is an essential regulator of *P. falciparum* asexual proliferation and host cell invasion genes with a dynamic genomic occupancy responsive to artemisinin stress

Abhishek Kanyal[1], Bhagyashree Deshmukh[1], Heledd Davies[2], D.V. Mamatharani[1], Dilsha Farheen[1], Moritz Treeck[2] and Krishanpal Karmodiya[1]*

[1]Department of Biology, Indian Institute of Science Education and Research, Dr. Homi Bhabha Road, Pashan, Pune 411008, Maharashtra, India

[2]Signalling in Apicomplexan Parasites Laboratory, The Francis Crick Institute, London, UK.

***Correspondence to:** krish@iiserpune.ac.in

Keywords: Malaria; *Plasmodium falciparum*; Histone deacetylase; PfHDAC1; Transcriptomics; Artemisinin resistance

Supplementary Material

PfHDAC1 is a regulator of the parasite intraerythrocytic development and governs the gene expression associated with vital biological functions

Having noted the invasion/proliferation boosting effects of PfHDAC1 overexpression we next decided to check the effect of catalytic inhibition of PfHDAC1. This was done as a proxy for a PfHDAC1 knock sideways/down which was unsuccessful. Class I HDACs have been well characterised for their role in cell cycle regulation in other systems [30]. HDAC1 is known for regulating expression of cyclins and cyclin-dependent kinases and is an integral factor governing cell cycle checkpoint dynamics in mammals [78].

We inhibited PfHDAC1 using continuous sublethal dosage (0.5 X IC_{50}) of romidepsin (a class I HDAC inhibitor with an IC_{50} for *Plasmodium falciparum* as 180nM) and followed the intraerythrocytic cell cycle progression via microscopic counts/examination at 8-hour intervals (starting at 6-hour post invasion mark). Inhibitor treatment was marked by delayed persistence of rings (22HPI), morphologically stunted trophozoites (30HPI) and a delayed schizont segmentation and merozoite egress (46HPI) and reinvasion (56HPI relative to cycle1) into subsequent IDC cycle2 (Supplementary Figure 3A and B). We observed a decrease in overall parasitaemia (11% in control and 7% upon romidepsin treatment) in the second intraerythrocytic developmental cycle (Supplementary Figure 3C). Thus, PfHDAC1 inhibition was shown to delay cell cycle progression compounded with defects in proper morphological development of parasites and reduction in proliferation over to the next cycle of the intraerythrocytic development.

We next investigated the effect of romidepsin (and entinostat) on the in-vivo stability of PfHDAC1. Early trophozoite stage parasites treated with 2XIC_{50} dosage of either entinostat (20µM) or romidepsin (360nM) for 8hr (20-28HPI) revealed lower levels of the protein upon

treatment with either inhibitor (Supplementary Figure 3D). Other studies in mammalian system have also hinted at the effect of romidepsin on the levels of HDAC1 [79]. Treatment of parasites with 50nM dihydroartemisinin antimalarial (which is also known to stall asexual parasite development) for 8 hours also resulted in a depletion of PfHDAC1 protein levels (Supplementary Figure 3D). Given that PfHDAC1 transcription stays fairly stable over this time-frame the depletion of PfHDAC1 protein levels in this condition cannot be accounted by developmental stalling alone and hint at possible effect of the inhibitors and artemisinin on PfHDAC1 stability [41].

We next investigated the transcriptional changes taking place in parasites upon PfHDAC1 inhibitor treatment. RNA sequencing (in biological triplicate) was performed on 21-24 HPI trophozoite stage parasites treated with romidepsin (360nM; 2XIC_{50} for 3 hrs). The choice of timepoint is at which PfHDAC1 levels have surged and stabilized and overall transcription increases [41]. A 3-hour pulse of 2X IC_{50} dosage was chosen (non-lethal) to record the early transcriptional effects of the inhibitor. We observed deregulation of 1906 genes (1227 upregulated and 679 gene downregulated; log2fold-change >= -1/1; p-value<=0.05, mean-count>=10) with PfHDAC1 inhibitor treatment (Supplementary Figure 3E; Supplementary Table 3). Among the biological processes upregulated were entry into host, microtubule-based locomotion, fatty acid metabolism and cell signaling (Supplementary Figure 3F). Downregulation of genes associated with critical biological processes like RNA splicing/metabolism, transcription by RNA polymerase II, translation, DNA damage repair, cell cycle, DNA replication and vesicle mediated transport among others was observed (Supplementary Figure 3G). Among the DNA replication associated gene set were Centrin-1 (PF3D7_0107000), anaphase promoting complex protein 1 (PF3D7_0728100), SMC protein 1 (PF3D7_1130700), DNA replication licencing factors MCM3 (PF3D7_0527000), MCM7 (PF3D7_0705400).

Upon comparison of differentially expressed genes upon romidepsin treatment with PfHDAC1 ChIP we observed a substantial overlap. 695 of the 1393 PfHDAC1 target genes (50% of total targets) were deregulated upon romidepsin treatment (Supplementary Figure 4A Right). The additional genes deregulated in romidepsin treatment may be a consequence of the off-target effects of romidepsin or downstream indirect effects of PfHDAC1 activity suppression. Direct targets of PfHDAC1 which got upregulated were associated with entry/exit into host cells, cytoskeletal organisation and response to unfolded protein. Direct targets which got downregulated upon romidepsin treatment were associated with DNA replication, RNA splicing, DNA geometric changes, DNA repair and cell cycle progress.

We additionally compared the our romidepsin treatment differential gene expression dataset the PfHDAC1 genetic depletion RNA sequencing by Huang et. al. 2020 [34]. A global analysis of the gene expression changes (among the common 5377 genes across the 2 datasets) revealed a weak positive correlation in differentially expressed genes (R= 0.21 pvalue<=2.2e-16) (Supplementary Figure 4B). The lack of a strong correlation between the two datasets could stem from additional off-target effects of romidepsin and parasite strain specific differences. We identified 1039 genes to be commonly upregulated, these were principally associated with microtubule-based movement, fatty acid biosynthesis, signal transduction, and response to heat shock. 441 genes were found to be commonly downregulated commonly and principally associated with vesicular transport, double-strand break repair, phosphorylation, cell cycle and DNA replication (Supplementary Figure 4C and Supplementary Table 4). Notably, treatment of parasites with romidepsin was found to enhance H3K9ac histone modification levels, which falls in line with the role of PfHDAC1 as a histone deacetylase (Supplementary Figure 4D).

Collectively these observations highlight the importance of PfHDAC1 activity in timely progression of the parasite IDC, optimal DNA replication and passage of infection to the consecutive cycle. PfHDAC1 inhibition in parasites was found to deregulate the expression of several crucial biological pathways including DNA replication, splicing, microtubule-based movement and signal transduction. Of these deregulated genes at least, half were direct genomic targets of PfHDAC1 in trophozoites.

Supplementary Figure Legends

Supplementary Figure 1: (A) IGV genome browser view of the PfHDAC1-2xFKBP-GFP genome sequencing (from pre-ChIP input genomic DNA) alignments (BAM) onto a modified PfHDAC1 reference sequence (includes the PfHDAC1 sequence fused with 2xFKBP, GFP and linker sequences). The alignments are colored in red or blue for forward and reverse strand mapping. (B) 1% agarose gel image of amplicons resolved from various PCR reactions using specific primer pairs in each case to confirm PfHDAC1-2xFKBP-GFP knock-in parasite line (versus a wildtype NF54 control line). A schematic of the primer binding on modified PfHDAC1 locus in the knock-in transgenic line is provided at top of gel image. (C) Confocal images of ring, trophozoite and schizont stage parasites from PfHDAC1-2xFKBP-GFP knock in parasite line. Tagged PfHDAC1 is evident in GFP panel while nuclei are stained with DAPI and parasite visualized in brightfield and merged for reference. (D) Western blotting image for protein abundance of 2xFKBP-GFP tagged PfHDAC1 in ring, trophozoite and schizont stage parasites. The modified PfHDAC1 is evident as a band at expected 100kDa size. α-actin probing is shown as a loading control. The image is representative of three biological replicates.

Supplementary Figure 2: (A) Spearman correlation heatmap of the input normalized ChIP datasets for the various stage time-points of ChIP experiment. Figures for ChIP-seq are prepared from two biological replicates. (B) Venn diagram of shared and unique PfHDAC1 gene targets across the three stages of asexual parasite development (C) Line plots depicting the growth trends of PfHDAC1-GFP-glmS overexpression parasites cultured under different dosage of glucosamine-HCl across 3 parasite invasion cycles. Image is representative of two biological replicates. (D) Giemsa-stained smear panel to comparing the parasite progression through the intraerythrocytic development cycle for GFP-glmS ctrl overexpression and PfHDAC1-GFP-glmS overexpression parasite lines. Images are representative of one biological replicate noted in three technical replicates. (E) Giemsa-stained smear comparing merozoite counts at 44HPI schizonts in PfHDAC1-GFP-glmS and GFP-glmS overexpression parasite lines. Images are representative of two biological replicates (30 schizonts were analysed per sample per replicate). (F) Plot depicting the count of merozoites from individual schizonts of PfHDAC1-GFP-glmS overexpression parasites cultured for 3 cycles under different glucosamine-HCl dosage and treated with 10µM E64 to arrest merozoite egress. Image is representative of two biological replicates.

Supplementary Figure 3: (A) Giemsa-stained smear panel to comparing the parasite progression through the intraerythrocytic development cycle upon mock and romidepsin treatment (0.5X IC_{50} or 90nM). Images taken at 8-hour intervals from 6HPI onwards. (B) Histograms to represent relative proportion of parasites from each of the three major stages (ring, trophozoite and schizont) in mock vs romidepsin treated parasite cultures. (C) Dot plot to represent the lower reinvasion rates and parasitemia progression in romidepsin treated parasite compared to mock control. (Statistical analysis used: Student's unpaired t-test; **** represent p-value <0.0001). Figure A-C are obtained from one biological experimental

replicate and three technical replicate reading for each datapoint. (D) Western blot for α-HDAC1 (top panel), α-HSP70 (2nd panel), α-BiP (3rd panel) and α-actin (bottom panel, loading control) under mock vs entinostat (20μM), romidepsin (360nM), and dihydroartemisinin (50nM) treatment through 20-28HPI (8hr treatment) parasites. Representative of two biological replicates. (E) Volcano plot representing the differential gene expression upon romidepsin treatment (2X IC_{50} 360nM for 3hr/21-24HPI) of parasite culture. Dot plots representing the biological processes (F) upregulated and (G) downregulated upon romidepsin treatment in parasites. The RNA seq data is from three biological replicates.

Supplementary Figure 4: (A Left) Venn diagrams representing the overlap of 24HPI trophozoite stage PfHDAC1 genomic targets (Green) with genes upregulated (Blue) or downregulated (Yellow) with romidepsin treatment. Log2FC cutoff is +/-1. (A Right) Venn diagrams representing the overlap of 24HPI trophozoite stage PfHDAC1 genomic targets (Green) with genes upregulated (Blue) or downregulated (Yellow) with romidepsin treatment. Log2FC cutoff is +/-0.5. Both venn diagrams are derived from three biological replicate RNA sequencing (360nM romidepsin for 3hr at 21-24HPI vs Mock). (B) Scatter Plot for gene deregulation upon romidepsin treatment in our dataset vs gene deregulated upon PfHDAC1 genetic depletion in Huang et. al., 2020; Cell Discovery. (C) Boxplots representing genes from biological pathways upregulated (Left) and downregulated (Right) with glucosamine mediated depletion of PfHDAC1-GFP-glmS. Data analyzed from Huang et. al., 2020; Cell Discovery. (D) Western blot for H3K9ac levels and actin (loading control) in mock vs romidepsin treated parasites (21-24HPI treatment; 360nM romidepsin). Image is representative of two biological replicates.

Supplementary Figure 5: (A-D) IGV genome browser tracks for PfKelch13 gene in PfHDAC1-GFP-glmS and GFP-glmS overexpression parasite lines created on PfKelch13 wildtype or mutant lines. (A) Highlighted region (pink) confirms presence of R539T mutation in the resistant parental line. The I543 locus (yellow highlight) (panel A) and C580 locus (purple highlight) (panel B) is wildtype in all lines tested. (C and D) Shared SNPs detected on the PfKelch13 locus (grey highlight) which potentially belong to isolates from the geographical origin. *Plasmodium falciparum* 3D7 genome and GTF version 59 is loaded as reference.

Supplementary Figure 6: (A) Netphos 3.1 analysis of PfHDAC1 amino acid sequence predicts CKII to be the kinase associated with S391, S397 and S440 phosphorylated residues. (B) FASTA format sequence of PfHDAC1 protein followed by the predicted post-translational modifications at key sites (C) Histogram representing the phosphorylation potential of serine, threonine and tyrosine residues across PfHDAC1 sequence. Serine 391 and 397 are boxed in black while serine 440 is boxed in blue for clarity. (D) Full Western blot (using α-GST antibody) for in vitro kinase activity assay using purified recombinant PfHDAC1 and PfCKII-α. The reaction mix components are as specified in the following table. The conc. of CKII-α inhibitor used was 6μM(+) and 12μM(++). The conc. of dihydroartemisinin was 100nM. Experiment was performed in two biological replicates. Western blot (using α-phospho Ser/Thr antibody for the same in vitro kinase activity assay as represented in right panel. The experiment was performed in two biological replicates.

Supplementary Table Legends

Supplementary Table 1: A table summarizing the ChIP-sequencing and RNA-sequencing experiments performed in this study with details on sample ID, strain and stage of parasite, treatment and duration (if any).

Supplementary Table 2: Details of PfHDAC1 ChIP peaks called by MACS2 software. The file has 5 tabs for the data from ring (10HPI), trophozoite (21HPI), trophozoite (24HPI), schizont (40HPI) and DHA treated 24HPI trophozoites. Each tab has a list of peaks (>=2fold-enrichment) and list of genes these peaks map onto.

Supplementary Table 3: Details of the DESeq2 differential gene expression analysis. The file has 4 tabs. Tab 1 and 2 relate to list of differentially expressed genes and the Deseq2 related metadata from PfHDAC1-GFP-glmS overexpression vs GFP-glmS control RNA-sequencing. Tab 3 and 4 relate to list of differentially expressed genes and the Deseq2 related metadata from romidepsin vs mock treatment RNA-sequencing.

Supplementary Table 4: Document enlisting the log2(foldchange) across genes deregulated upon romidepsin treatment in this study vs PfHDAC1 depletion in Huang et. al. 2020 (Tab 1). Common genes upregulated (Tab 2) and downregulated (Tab 3) between both datasets are listed along with the Gene Ontology for the respective gene sets (Tab 4 and 5).

Supplementary Table 1:

ChIP-sequencing Sample Details

SN	Sample Name	Strain	Stage	Treatment
1	PfHDAC1_Ring_ChIP1	NF54	Ring 10HPI	NA
2	PfHDAC1_Ring_ChIP2	NF54	Ring 10HPI	NA
3	PfHDAC1_Ring_Input	NF54	Ring 10HPI	NA
4	PfHDAC1_Troph21HPI_ChIP1	NF54	Troph 21HPI	NA
5	PfHDAC1_Troph21HPI_ChIP2	NF54	Troph 21HPI	NA
6	PfHDAC1_Troph21HPI_Input	NF54	Troph 21HPI	NA
7	PfHDAC1_Troph24HPI_ChIP1	NF54	Troph 24HPI	NA
8	PfHDAC1_Troph24HPI_ChIP2	NF54	Troph 24HPI	NA
9	PfHDAC1_Troph24HPI_Input	NF54	Troph 24HPI	NA
10	PfHDAC1_Troph21-24HPI_DHA_Chip1	NF54	Troph 24HPI	100nM DHA/3hr
11	PfHDAC1_Troph21-24HPI_DHA_Chip2	NF54	Troph 24HPI	100nM DHA/3hr
12	PfHDAC1_Troph21-24HPI_DHA_Input	NF54	Troph24HPI	100nM DHA/3hr

RNA-sequencing Sample Details

SN	Sample Name	Strain	Stage	Treatment
1	Ctrl_Rep1	3D7	Troph (21-24HPI)	DMSO Mock/3hr
2	Ctrl_Rep2	3D7	Troph (21-24HPI)	DMSO Mock/3hr
3	Ctrl_Rep3	3D7	Troph (21-24HPI)	DMSO Mock/3hr
4	Rom_Rep1	3D7	Troph (21-24HPI)	360nM Romidepsin/3hr
5	Rom_Rep2	3D7	Troph (21-24HPI)	360nM Romidepsin/3hr
6	Rom_Rep3	3D7	Troph (21-24HPI)	360nM Romidepsin/3hr
7	PfHDAC1_OE_Rep1	Cam3.I_rev/MRA1252	Troph (21-24HPI)	NA
8	PfHDAC1_OE_Rep2	Cam3.I_rev/MRA1252	Troph (21-24HPI)	NA
9	PfHDAC1_OE_Rep3	Cam3.I_rev/MRA1252	Troph(21-24HPI)	NA
10	GFP_OE_Rep1	Cam3.I_rev/MRA1252	Troph (21-24HPI)	NA
11	GFP_OE_Rep2	Cam3.I_rev/MRA1252	Troph (21-24HPI)	NA
12	GFP_OE_Rep3	Cam3.I_rev/MRA1252	Troph (21-24HPI)	NA

References for supplementary results text

1. WHO (2022) *World Malaria Report 2022*, World Health Organization.
2. Ross, A., Maire, N., Molineaux, L. & Smith, T. (2006) An epidemiologic model of severe morbidity and mortality caused by Plasmodium falciparum, *The American Journal of Tropical Medicine Hygiene.* **75**, 63-73.
3. Müller, I. B. & Hyde, J. E. (2010) Antimalarial drugs: modes of action and mechanisms of parasite resistance, *Future Microbiology.* **5**, 1857-1873.
4. WHO (2016) Artemisinin and artemisinin-based combination therapy resistance: status report in, World Health Organization,
5. Ariey, F., Witkowski, B., Amaratunga, C., Beghain, J., Langlois, A.-C., Khim, N., Kim, S., Duru, V., Bouchier, C. & Ma, L. (2014) A molecular marker of artemisinin-resistant Plasmodium falciparum malaria, *Nature.* **505**, 50-55.
6. Birnbaum, J., Scharf, S., Schmidt, S., Jonscher, E., Hoeijmakers, W. A. M., Flemming, S., Toenhake, C. G., Schmitt, M., Sabitzki, R. & Bergmann, B. (2020) A Kelch13-defined endocytosis pathway mediates artemisinin resistance in malaria parasites, *Science.* **367**, 51-59.
7. Demas, A. R., Sharma, A. I., Wong, W., Early, A. M., Redmond, S., Bopp, S., Neafsey, D. E., Volkman, S. K., Hartl, D. L. & Wirth, D. F. (2018) Mutations in Plasmodium falciparum actin-binding protein coronin confer reduced artemisinin susceptibility, *Proceedings of the National Academy of Sciences.* **115**, 12799-12804.
8. Mbengue, A., Bhattacharjee, S., Pandharkar, T., Liu, H., Estiu, G., Stahelin, R. V., Rizk, S. S., Njimoh, D. L., Ryan, Y. & Chotivanich, K. (2015) A molecular mechanism of artemisinin resistance in Plasmodium falciparum malaria, *Nature.* **520**, 683-687.
9. Mok, S., Ashley, E. A., Ferreira, P. E., Zhu, L., Lin, Z., Yeo, T., Chotivanich, K., Imwong, M., Pukrittayakamee, S. & Dhorda, M. (2015) Population transcriptomics of human malaria parasites reveals the mechanism of artemisinin resistance, *Science.* **347**, 431-435.
10. Mok, S., Stokes, B. H., Gnädig, N. F., Ross, L. S., Yeo, T., Amaratunga, C., Allman, E., Solyakov, L., Bottrill, A. R. & Tripathi, J. (2021) Artemisinin-resistant K13 mutations rewire Plasmodium falciparum's intra-erythrocytic metabolic program to enhance survival, *Nature Communications.* **12**, 1-15.
11. Bozdech, Z., Llinás, M., Pulliam, B. L., Wong, E. D., Zhu, J., DeRisi, J. L. & Ward, G. (2003) The transcriptome of the intraerythrocytic developmental cycle of Plasmodium falciparum, *PLoS Biology.* **1**, e5.
12. Le Roch, K. G., Johnson, J. R., Florens, L., Zhou, Y., Santrosyan, A., Grainger, M., Yan, S. F., Williamson, K. C., Holder, A. A. & Carucci, D. J. (2004) Global analysis of transcript and protein levels across the Plasmodium falciparum life cycle, *Genome Research.* **14**, 2308-2318.
13. Gardner, M. J., Hall, N., Fung, E., White, O., Berriman, M., Hyman, R. W., Carlton, J. M., Pain, A., Nelson, K. E. & Bowman, S. (2002) Genome sequence of the human malaria parasite Plasmodium falciparum, *Nature.* **419**, 498-511.
14. Hollin, T. & Le Roch, K. G. (2020) From genes to transcripts, a tightly regulated journey in plasmodium, *Frontiers in Cellular Infection Microbiology.* **10**, 618454.
15. Cui, L., Miao, J., Furuya, T., Li, X., Su, X.-z. & Cui, L. (2007) PfGCN5-mediated histone H3 acetylation plays a key role in gene expression in Plasmodium falciparum, *Eukaryotic cell.* **6**, 1219-1227.
16. Tabassum, W., Bhattacharyya, S., Varunan, S. M. & Bhattacharyya, M. K. (2021) Febrile temperature causes transcriptional downregulation of Plasmodium falciparum Sirtuins through Hsp90-dependent epigenetic modification, *Molecular Microbiology.* **115**, 1025-1038.
17. Tonkin, C. J., Carret, C. K., Duraisingh, M. T., Voss, T. S., Ralph, S. A., Hommel, M., Duffy, M. F., Silva, L. M. d., Scherf, A. & Ivens, A. (2009) Sir2 paralogues cooperate to

regulate virulence genes and antigenic variation in Plasmodium falciparum, *PLoS Biology.* **7**, e1000084.
18. Engel, J. A., Jones, A. J., Avery, V. M., Sumanadasa, S. D., Ng, S. S., Fairlie, D. P., Adams, T. S. & Andrews, K. T. (2015) Profiling the anti-protozoal activity of anti-cancer HDAC inhibitors against Plasmodium and Trypanosoma parasites, *International Journal for Parasitology: Drugs Drug Resistance.* **5**, 117-126.
19. Bushell, E., Gomes, A. R., Sanderson, T., Anar, B., Girling, G., Herd, C., Metcalf, T., Modrzynska, K., Schwach, F. & Martin, R. E. (2017) Functional profiling of a Plasmodium genome reveals an abundance of essential genes, *Cell.* **170**, 260-272. e8.
20. Zhang, M., Wang, C., Otto, T. D., Oberstaller, J., Liao, X., Adapa, S. R., Udenze, K., Bronner, I. F., Casandra, D. & Mayho, M. (2018) Uncovering the essential genes of the human malaria parasite Plasmodium falciparum by saturation mutagenesis, *Science.* **360**.
21. Elbadawi, M. A. A., Awadalla, M. K. A., Hamid, M. M. A., Mohamed, M. A. & Awad, T. A. (2015) Valproic acid as a potential inhibitor of Plasmodium falciparum histone deacetylase 1 (PfHDAC1): an in silico approach, *International Journal of Molecular Sciences.* **16**, 3915-3931.
22. Hansen, F. K., Sumanadasa, S. D., Stenzel, K., Duffy, S., Meister, S., Marek, L., Schmetter, R., Kuna, K., Hamacher, A. & Mordmüller, B. (2014) Discovery of HDAC inhibitors with potent activity against multiple malaria parasite life cycle stages, *European Journal of Medicinal Chemistry.* **82**, 204-213.
23. Hesping, E., Skinner-Adams, T. S., Fisher, G. M., Kurz, T. & Andrews, K. T. (2020) An ELISA method to assess HDAC inhibitor-induced alterations to P. falciparum histone lysine acetylation, *International Journal for Parasitology: Drugs Drug Resistance.* **14**, 249-256.
24. Wheatley, N. C., Andrews, K. T., Tran, T. L., Lucke, A. J., Reid, R. C. & Fairlie, D. P. (2010) Antimalarial histone deacetylase inhibitors containing cinnamate or NSAID components, *Bioorganic Medicinal Chemistry Letters.* **20**, 7080-7084.
25. Dovey, O. M., Foster, C. T. & Cowley, S. M. (2010) Histone deacetylase 1 (HDAC1), but not HDAC2, controls embryonic stem cell differentiation, *Proceedings of the National Academy of Sciences.* **107**, 8242-8247.
26. Kulka, L. A. M., Fangmann, P.-V., Panfilova, D. & Olzscha, H. (2020) Impact of HDAC inhibitors on protein quality control systems: consequences for precision medicine in malignant disease, *Frontiers in Cell Developmental Biology.* **8**, 425.
27. Milutinovic, S., Zhuang, Q. & Szyf, M. (2002) Proliferating cell nuclear antigen associates with histone deacetylase activity, integrating DNA replication and chromatin modification, *Journal of Biological Chemistry.* **277**, 20974-20978.
28. Oh, M., Choi, I.-K. & Kwon, H. J. (2008) Inhibition of histone deacetylase1 induces autophagy, *Biochemical Biophysical Research Communications.* **369**, 1179-1183.
29. Thurn, K. T., Thomas, S., Raha, P., Qureshi, I. & Munster, P. N. (2013) Histone deacetylase regulation of ATM-mediated DNA damage signaling, *Molecular Cancer Therapeutics.* **12**, 2078-2087.
30. Wilting, R. H., Yanover, E., Heideman, M. R., Jacobs, H., Horner, J., Van Der Torre, J., DePinho, R. A. & Dannenberg, J. H. (2010) Overlapping functions of Hdac1 and Hdac2 in cell cycle regulation and haematopoiesis, *The EMBO Journal.* **29**, 2586-2597.
31. Mok, S., Imwong, M., Mackinnon, M. J., Sim, J., Ramadoss, R., Yi, P., Mayxay, M., Chotivanich, K., Liong, K.-Y. & Russell, B. (2011) Artemisinin resistance in Plasmodium falciparum is associated with an altered temporal pattern of transcription, *BMC Genomics.* **12**, 1-14.
32. Hanisch, D., Krumm, A., Diehl, T., Stork, C. M., Dejung, M., Butter, F., Kim, E., Brenner, W., Fritz, G., Hofmann, T. G. & Disease (2022) Class I HDAC overexpression promotes temozolomide resistance in glioma cells by regulating RAD18 expression, *Cell Death.* **13**, 293.

33. Lai, Q.-y., He, Y.-z., Peng, X.-w., Zhou, X., Liang, D., Wang, L. & Signaling (2019) Histone deacetylase 1 induced by neddylation inhibition contributes to drug resistance in acute myelogenous leukemia, *Cell Communication.* **17**, 1-14.
34. Huang, Z., Li, R., Tang, T., Ling, D., Wang, M., Xu, D., Sun, M., Zheng, L., Zhu, F. & Min, H. (2020) A novel multistage antiplasmodial inhibitor targeting Plasmodium falciparum histone deacetylase 1, *Cell Discovery.* **6**, 1-15.
35. Mukherjee, P., Pradhan, A., Shah, F., Tekwani, B. L. & Avery, M. A. (2008) Structural insights into the Plasmodium falciparum histone deacetylase 1 (PfHDAC-1): A novel target for the development of antimalarial therapy, *Bioorganic Medicinal Chemistry.* **16**, 5254-5265.
36. Patel, V., Mazitschek, R., Coleman, B., Nguyen, C., Urgaonkar, S., Cortese, J., Barker Jr, R. H., Greenberg, E., Tang, W. & Bradner, J. E. (2009) Identification and characterization of small molecule inhibitors of a class I histone deacetylase from Plasmodium falciparum, *Journal of Medicinal Chemistry.* **52**, 2185-2187.
37. Birnbaum, J., Flemming, S., Reichard, N., Soares, A. B., Mesén-Ramírez, P., Jonscher, E., Bergmann, B. & Spielmann, T. (2017) A genetic system to study Plasmodium falciparum protein function, *Nature Methods.* **14**, 450-456.
38. Le Roch, K. G., Zhou, Y., Blair, P. L., Grainger, M., Moch, J. K., Haynes, J. D., De la Vega, P., Holder, A. A., Batalov, S. & Carucci, D. J. (2003) Discovery of gene function by expression profiling of the malaria parasite life cycle, *Science.* **301**, 1503-1508.
39. Howick, V. M., Russell, A. J., Andrews, T., Heaton, H., Reid, A. J., Natarajan, K., Butungi, H., Metcalf, T., Verzier, L. H. & Rayner, J. C. (2019) The Malaria Cell Atlas: Single parasite transcriptomes across the complete Plasmodium life cycle, *Science.* **365**, eaaw2619.
40. Brochet, M. & Billker, O. (2016) Calcium signalling in malaria parasites, *Journal of Molecular Microbiology.* **100**, 397-408.
41. Toenhake, C. G., Fraschka, S. A.-K., Vijayabaskar, M. S., Westhead, D. R., van Heeringen, S. J. & Bártfai, R. (2018) Chromatin accessibility-based characterization of the gene regulatory network underlying Plasmodium falciparum blood-stage development, *Cell Host Microbe.* **23**, 557-569. e9.
42. Beeson, J. G., Drew, D. R., Boyle, M. J., Feng, G., Fowkes, F. J. & Richards, J. S. (2016) Merozoite surface proteins in red blood cell invasion, immunity and vaccines against malaria, *FEMS Microbiology Reviews.* **40**, 343-372.
43. Counihan, N. A., Kalanon, M., Coppel, R. L. & de Koning-Ward, T. F. (2013) Plasmodium rhoptry proteins: why order is important, *Trends in Parasitology.* **29**, 228-236.
44. Amlabu, E., Mensah-Brown, H., Nyarko, P. B., Akuh, O.-a., Opoku, G., Ilani, P., Oyagbenro, R., Asiedu, K., Aniweh, Y. & Awandare, G. A. (2018) Functional characterization of Plasmodium falciparum surface-related antigen as a potential blood-stage vaccine target, *The Journal of Infectious Diseases.* **218**, 778-790.
45. Kumar, S., Kumar, M., Ekka, R., Dvorin, J. D., Paul, A. S., Madugundu, A. K., Gilberger, T., Gowda, H., Duraisingh, M. T. & Keshava Prasad, T. (2017) PfCDPK1 mediated signaling in erythrocytic stages of Plasmodium falciparum, *Nature Communications.* **8**, 63.
46. Wilde, M.-L., Triglia, T., Marapana, D., Thompson, J. K., Kouzmitchev, A. A., Bullen, H. E., Gilson, P. R., Cowman, A. F. & Tonkin, C. J. (2019) Protein kinase A is essential for invasion of Plasmodium falciparum into human erythrocytes, *MBio.* **10**, e01972-19.
47. Straimer, J., Gnädig, N. F., Witkowski, B., Amaratunga, C., Duru, V., Ramadani, A. P., Dacheux, M., Khim, N., Zhang, L. & Lam, S. (2015) K13-propeller mutations confer artemisinin resistance in Plasmodium falciparum clinical isolates, *Science.* **347**, 428-431.
48. Josling, G. A., Williamson, K. C. & Llinás, M. (2018) Regulation of sexual commitment and gametocytogenesis in malaria parasites, *Annual Reviews of Microbiology.* **72**, 501-519.
49. Lasonder, E., Rijpma, S. R., van Schaijk, B. C., Hoeijmakers, W. A., Kensche, P. R., Gresnigt, M. S., Italiaander, A., Vos, M. W., Woestenenk, R. & Bousema, T. (2016) Integrated

transcriptomic and proteomic analyses of P. falciparum gametocytes: molecular insight into sex-specific processes and translational repression, *Nucleic Acids Research.* **44**, 6087-6101.
50. Prieto, J. H., Koncarevic, S., Park, S. K., Yates III, J. & Becker, K. (2008) Large-scale differential proteome analysis in Plasmodium falciparum under drug treatment, *PLoS One.* **3**, e4098.
51. Siwo, G. H., Smith, R. S., Tan, A., Button-Simons, K. A., Checkley, L. A. & Ferdig, M. T. (2015) An integrative analysis of small molecule transcriptional responses in the human malaria parasite Plasmodium falciparum, *BMC Genomics.* **16**, 1-17.
52. Zhu, L., van der Pluijm, R. W., Kucharski, M., Nayak, S., Tripathi, J., White, N. J., Day, N. P., Faiz, A., Phyo, A. P. & Amaratunga, C. (2022) Artemisinin resistance in the malaria parasite, Plasmodium falciparum, originates from its initial transcriptional response, *Communications Biology.* **5**, 274.
53. Breglio, K. F., Amato, R., Eastman, R., Lim, P., Sa, J. M., Guha, R., Ganesan, S., Dorward, D. W., Klumpp-Thomas, C. & McKnight, C. (2018) A single nucleotide polymorphism in the Plasmodium falciparum atg18 gene associates with artemisinin resistance and confers enhanced parasite survival under nutrient deprivation, *Malaria Journal.* **17**, 1-16.
54. Pflum, M. K. H., Tong, J. K., Lane, W. S. & Schreiber, S. L. (2001) Histone deacetylase 1 phosphorylation promotes enzymatic activity and complex formation, *Journal of Biological Chemistry.* **276**, 47733-47741.
55. Pease, B. N., Huttlin, E. L., Jedrychowski, M. P., Talevich, E., Harmon, J., Dillman, T., Kannan, N., Doerig, C., Chakrabarti, R. & Gygi, S. P. (2013) Global analysis of protein expression and phosphorylation of three stages of Plasmodium falciparum intraerythrocytic development, *Journal of Proteome Research.* **12**, 4028-4045.
56. Treeck, M., Sanders, J. L., Elias, J. E. & Boothroyd, J. C. (2011) The phosphoproteomes of Plasmodium falciparum and Toxoplasma gondii reveal unusual adaptations within and beyond the parasites' boundaries, *Cell Host Microbe.* **10**, 410-419.
57. Draney, C., Austin, M. C., Leifer, A. H., Smith, C. J., Kener, K. B., Aitken, T. J., Hess, K. H., Haines, A. C., Lett, E. & Hernandez-Carretero, A. (2018) HDAC1 overexpression enhances β-cell proliferation by down-regulating Cdkn1b/p27, *Biochemical Journal.* **475**, 3997-4010.
58. Kawai, H., Li, H., Avraham, S., Jiang, S. & Avraham, H. K. (2003) Overexpression of histone deacetylase HDAC1 modulates breast cancer progression by negative regulation of estrogen receptor α, *International Journal of Cancer.* **107**, 353-358.
59. Kakiuchi, A., Kakuki, T., Ohwada, K., Kurose, M., Kondoh, A., Obata, K., Nomura, K., Miyata, R., Kaneko, Y. & Konno, T. (2021) HDAC inhibitors suppress the proliferation, migration and invasiveness of human head and neck squamous cell carcinoma cells via p63-mediated tight junction molecules and p21-mediated growth arrest, *Oncology Reports.* **45**, 1-12.
60. Lasonder, E., Green, J. L., Grainger, M., Langsley, G. & Holder, A. A. (2015) Extensive differential protein phosphorylation as intraerythrocytic Plasmodium falciparum schizonts develop into extracellular invasive merozoites, *Proteomics.* **15**, 2716-2729.
61. Lyons, F. M., Gabriela, M., Tham, W.-H. & Dietrich, M. H. (2022) Plasmodium 6-cysteine proteins: functional diversity, transmission-blocking antibodies and structural scaffolds, *Frontiers in Cellular Infection Microbiology.* **12**, 945924.
62. Gupta, A., Thiruvengadam, G. & Desai, S. A. (2015) The conserved clag multigene family of malaria parasites: essential roles in host–pathogen interaction, *Drug Resistance Updates.* **18**, 47-54.
63. Counihan, N. A., Chisholm, S. A., Bullen, H. E., Srivastava, A., Sanders, P. R., Jonsdottir, T. K., Weiss, G. E., Ghosh, S., Crabb, B. S. & Creek, D. J. (2017) Plasmodium falciparum parasites deploy RhopH2 into the host erythrocyte to obtain nutrients, grow and replicate, *Elife.* **6**, e23217.

64. Pasternak, M., Verhoef, J. M., Wong, W., Triglia, T., Mlodzianoski, M. J., Geoghegan, N., Evelyn, C., Wardak, A. Z., Rogers, K. & Cowman, A. F. (2022) RhopH2 and RhopH3 export enables assembly of the RhopH complex on P. falciparum-infected erythrocyte membranes, *Communications Biology.* **5**, 333.
65. Richard, D., MacRaild, C. A., Riglar, D. T., Chan, J.-A., Foley, M., Baum, J., Ralph, S. A., Norton, R. S. & Cowman, A. F. (2010) Interaction between Plasmodium falciparum apical membrane antigen 1 and the rhoptry neck protein complex defines a key step in the erythrocyte invasion process of malaria parasites, *Journal of Biological Chemistry.* **285**, 14815-14822.
66. Srinivasan, P., Beatty, W. L., Diouf, A., Herrera, R., Ambroggio, X., Moch, J. K., Tyler, J. S., Narum, D. L., Pierce, S. K. & Boothroyd, J. C. (2011) Binding of Plasmodium merozoite proteins RON2 and AMA1 triggers commitment to invasion, *Proceedings of the National Academy of Sciences.* **108**, 13275-13280.
67. Weiss, G. E., Gilson, P. R., Taechalertpaisarn, T., Tham, W.-H., de Jong, N. W., Harvey, K. L., Fowkes, F. J., Barlow, P. N., Rayner, J. C. & Wright, G. J. (2015) Revealing the sequence and resulting cellular morphology of receptor-ligand interactions during Plasmodium falciparum invasion of erythrocytes, *PLoS Pathogens.* **11**, e1004670.
68. Li, W., Mo, W., Shen, D., Sun, L., Wang, J., Lu, S., Gitschier, J. M. & Zhou, B. (2005) Yeast model uncovers dual roles of mitochondria in the action of artemisinin, *PLoS Genetics.* **1**, e36.
69. Peatey, C. L., Chavchich, M., Chen, N., Gresty, K. J., Gray, K.-A., Gatton, M. L., Waters, N. C. & Cheng, Q. (2015) Mitochondrial membrane potential in a small subset of artemisinin-induced dormant Plasmodium falciparum parasites in vitro, *The Journal of Infectious Diseases.* **212**, 426-434.
70. Gupta, D. K., Patra, A. T., Zhu, L., Gupta, A. P. & Bozdech, Z. (2016) DNA damage regulation and its role in drug-related phenotypes in the malaria parasites, *Scientific Reports.* **6**, 23603.
71. Portugaliza, H. P., Miyazaki, S., Geurten, F. J., Pell, C., Rosanas-Urgell, A., Janse, C. J. & Cortes, A. (2020) Artemisinin exposure at the ring or trophozoite stage impacts Plasmodium falciparum sexual conversion differently, *Elife.* **9**, e60058.
72. Halbert, J., Ayong, L., Equinet, L., Le Roch, K., Hardy, M., Goldring, D., Reininger, L., Waters, N., Chakrabarti, D. & Doerig, C. (2010) A Plasmodium falciparum transcriptional cyclin-dependent kinase-related kinase with a crucial role in parasite proliferation associates with histone deacetylase activity, *Eukaryotic Cell.* **9**, 952-959.
73. Tham, W.-H., Lim, N. T., Weiss, G. E., Lopaticki, S., Ansell, B. R., Bird, M., Lucet, I., Dorin-Semblat, D., Doerig, C. & Gilson, P. R. (2015) Plasmodium falciparum adhesins play an essential role in signalling and activation of invasion into human erythrocytes, *PLoS Pathogens.* **11**, e1005343.
74. Quinlan, A. R. & Hall, I. M. (2010) BEDTools: a flexible suite of utilities for comparing genomic features, *Bioinformatics.* **26**, 841-842.
75. Thorvaldsdóttir, H., Robinson, J. T. & Mesirov, J. P. (2013) Integrative Genomics Viewer (IGV): high-performance genomics data visualization and exploration, *Briefings in Bioinformatics.* **14**, 178-192.
76. Ramírez, F., Dündar, F., Diehl, S., Grüning, B. A. & Manke, T. (2014) deepTools: a flexible platform for exploring deep-sequencing data, *Nucleic Acids Research.* **42**, W187-W191.
77. Aurrecoechea, C., Brestelli, J., Brunk, B. P., Dommer, J., Fischer, S., Gajria, B., Gao, X., Gingle, A., Grant, G. & Harb, O. S. (2009) PlasmoDB: a functional genomic database for malaria parasites, *Nucleic Acids Research.* **37**, D539-D543.
78. Telles, E. & Seto, E. (2012) Modulation of cell cycle regulators by HDACs, *Frontiers in Bioscience.* **4**, 831.

79. Valdez, B., Brammer, J., Li, Y., Murray, D., Liu, Y., Hosing, C., Nieto, Y., Champlin, R. & Andersson, B. (2015) Romidepsin targets multiple survival signaling pathways in malignant T cells, *Blood Cancer Journal.* **5**, e357-e357.
